# CAF-1 and Rtt101p function within the replication-coupled chromatin assembly network to promote H4 K16ac, preventing ectopic silencing

**DOI:** 10.1371/journal.pgen.1009226

**Published:** 2020-12-07

**Authors:** Tiffany J. Young, Yi Cui, Claire Pfeffer, Emilie Hobbs, Wenjie Liu, Joseph Irudayaraj, Ann L. Kirchmaier

**Affiliations:** 1 Department of Biochemistry, Purdue University, West Lafayette, Indiana, United States of America; 2 Purdue University Center for Cancer Research, West Lafayette, Indiana, United States of America; 3 Bindley Bioscience Center, Purdue University, West Lafayette, Indiana, United States of America; 4 Department of Agricultural and Biological Engineering, Purdue University, West Lafayette, Indiana, United States of America; 5 Department of Bioengineering, Cancer Center at Illinois, Micro and Nanotechnology Laboratory, University of Illinois at Urbana Champaign, Urbana, Illinois, United States of America; Institute of Functional Epigenetics, GERMANY

## Abstract

Replication-coupled chromatin assembly is achieved by a network of alternate pathways containing different chromatin assembly factors and histone-modifying enzymes that coordinate deposition of nucleosomes at the replication fork. Here we describe the organization of a CAF-1-dependent pathway in *Saccharomyces cerevisiae* that regulates acetylation of histone H4 K16. We demonstrate factors that function in this CAF-1-dependent pathway are important for preventing establishment of silenced states at inappropriate genomic sites using a crippled *HMR* locus as a model, while factors specific to other assembly pathways do not. This CAF-1-dependent pathway required the cullin Rtt101p, but was functionally distinct from an alternate pathway involving Rtt101p-dependent ubiquitination of histone H3 and the chromatin assembly factor Rtt106p. A major implication from this work is that cells have the inherent ability to create different chromatin modification patterns during DNA replication via differential processing and deposition of histones by distinct chromatin assembly pathways within the network.

## Author summary

Replication-coupled chromatin assembly occurs via a network of alternate pathways through which histones are processed, and chromatin is disassembled in front of the replication fork, then reassembled behind the fork to ensure the inheritance of appropriate epigenetic states. Yet, despite being essential for maintaining cell identity across cell generations, the organization of this network and what distinct functions individual pathways within this network may play has remained poorly understood. Here, we have used molecular genetic and live cell protein-protein interaction strategies to probe this network. We highlight a CAF-1-dependent pathway with a unique role in regulating histone H4 K16ac to prevent the establishment of epigenetically silenced states at ectopic sites. We also discovered the cullin Rtt101p functions in this CAF-1-dependent pathway in a manner distinct from Rtt101p’s role in promoting chromatin assembly along a Rtt106p-dependent pathway via ubiquitination of histone H3. Our findings illustrate that cells use alternate chromatin assembly pathways within the network during DNA replication to create distinct chromatin modification patterns. These patterns, in turn, influence the probability of establishing new epigenetic states.

## Introduction

Replication-coupled chromatin assembly is a multi-step, multi-pathway process coordinated by histone modifying proteins, histone chaperones, and replication factors. In *Saccharomyces cerevisiae*, the chromatin assembly factors Asf1p, Rtt106p, Hif1p, and the CAF-1 complex, consisting of Cac1p, Cac2p, and Cac3p, coordinate the assembly of H3-H4 into nucleosomes on newly synthesized DNA [1–5]. In humans the functions of these proteins seem mainly conserved as homologs of CAF-1, Asf1p, and Hif1p exist and are respectively named CAF-1, ASF1A/ASF1B, and NASP [6–9]. An ortholog of Rtt106p, Daxx, is also present in humans, but has likely diverged functionally. Daxx contains a Rtt106p-like acidic domain and acts as a H3-H4 histone chaperone, but current evidence shows that Daxx binds to the mammalian replication-independent deposition H3 histone variant H3.3, rather than the replication-coupled variant H3.1, and functions only in replication-independent chromatin assembly [4,10]. This is in contrast to Rtt106p, which uses the sole yeast H3 variant for both replication-dependent and replication-independent chromatin assembly [11,12]. Why multiple histone deposition pathways exist and how they are regulated during replication is unclear. Similarly, our understanding of the interactions that occur within this network of replication-coupled H3-H4 nucleosome assembly pathways, how these interactions are regulated, and, in turn, influence histone modification patterns remains limited. However, defects in these pathways do result in altered histone modification patterns across the genome, defects in epigenetic processes, and altered responses to a variety of stressors ranging from oxidative stress to DNA damage [13–18].

During replication-coupled chromatin assembly, evidence is mounting that chromatin assembly factors can promote step-specific histone modifications, and these histone modifications may then help direct histones to specific assembly pathways within the network via modulating protein-protein interactions. In replication-coupled chromatin assembly, Asf1p and CAF-1 bind to histone H3-H4 dimers, whereas Rtt106p has been reported to bind to H3-H4 dimers as well as (H3-H4)_2_ tetramers [19–27]. In budding yeast, newly synthesized H3 histones are acetylated at K56 in S phase by the acetyltransferase Rtt109p, which requires H3-H4 to be in a complex with Asf1p for the acetylation event to occur [28–30]. The loss of H3 K56 acetylation, H3 K56ac, results in a decrease in the amount of H3 that co-precipitates with Cac2p and Rtt106p *in vivo* [4,23], and H3 K56ac-H4 binds to CAF-1 and Rtt106p with a higher affinity than unacetylated H3-H4 *in vitro* [4,21,31]. Thus, H3 K56ac promotes the interaction between H3-H4 and CAF-1 as well as between H3-H4 and Rtt106p. These findings are consistent with Asf1p and CAF-1 only having partially overlapping functions [32], and support a model in which Asf1p acts upstream of CAF-1 and Rtt106p during replication-coupled chromatin assembly, and in which H3 K56ac promotes transfer of histones H3-H4 from Asf1p to CAF-1 or Rtt106p. Thus, Asf1p, Rtt109p and H3 K56ac are important features of CAF-1- as well as Rtt106p-dependent chromatin assembly pathways in yeast. In humans, the transfer of H3-H4 from Asf1 to CAF-1 appears to be conserved [6,7], but whether H3 K56ac can promote interactions between H3-H4 and CAF-1 in humans is unclear [33].

In addition to being acetylated, H3 is ubiquitinated in a *RTT101*-dependent manner primarily at K122, but also at K121 and K125 during S phase [34]. Co-precipitation analyses show that the presence of ubiquitinated H3 decreases binding of Asf1p to H3-H4, and increases binding of H3-H4 to Rtt106p, but does not alter binding of H3-H4 to CAF-1 in yeast *in vivo* [34]. These data are consistent with the prediction that H3 ubiquitination promotes the transfer of H3-H4 to Rtt106p from Asf1p, but not to CAF-1. In contrast, similar co-precipitation analyses performed in human cells demonstrated that depletion of the Cul4 E3 ubiquitin ligase results in decreased association of H3 with both p150, the Cac1p homolog, and Daxx, implying histone ubiquitination may serve to regulate chromatin assembly pathway usage in mammals as well [34], but the reason for these differences between organisms has been unclear. In yeast, either CAF-1 or Rtt106p can deposit histones onto newly synthesized DNA [1,4]. However, as Cac2p and Rtt106p co-precipitate *in vitro* and *in vivo* [1,35], it is possible that H3-H4 might also be transferred between CAF-1 and Rtt106p. Neither *ASF1*, *CAC1*, nor *RTT106* are essential in budding yeast, and double or triple mutant combinations are also viable [1,4,36,37]. Thus, alternative pathways must function to support packaging of newly replicated DNA in these contexts.

Like Asf1p, CAF-1, and Rtt106p, Hif1p has been implicated as a histone H3-H4 chromatin assembly factor [5], but its relationship to other factors within the replication-coupled chromatin assembly network is less well characterized. Hif1p/NASP uses distinct mechanisms to bind either H3-H4 tetramers or H2A-H2B dimers, and Hif1p can also bind to octamers *in vitro* [38,39]. Similar to Hif1p, NASP can bind H2A-H2B dimers, H3-H4 dimers, or sNASP (somatic NASP) can dimerize and bind H3-H4 tetramers, as well as interact with ASF1A/B [38,40–42]. In yeast, Hif1p is found in the NuB4 complex with acetyltransferase Hat1p and Hat2p [5,43] via an interaction with Hat2p [42]. Hat1p plus Hat2p make up the HAT-B complex, which acetylates newly synthesized histones on H4 K5 and 12 [44–46]. Asf1p/H3/H4 interacts with HAT-B or NuB4 *in vitro* via H3-H4 contacts [42] and the stability of interactions between Asf1p and HAT-B or NuB4 *in vivo* require Hat2p [47]. Although both HAT-B and NuB4 form complexes with Asf1p/H3/H4 in yeast and in human cells [41,42,47], their relationship with respect to functioning upstream or in parallel to CAF-1 and Rtt106p-mediated chromatin assembly pathways, plus the extent to which they function in Asf1p-independent pathway(s) remain poorly understood. How H3 K56ac and H3 K122ub affect interactions between NuB4 and either Asf1p or H3-H4 is also unclear. However, loss of *RTT109* does not abrogate the association between Hat2p and Asf1p, indicating that H3 K56ac is not required for this interaction [41].

Acetylation of H4 K16, H4 K16ac, by SAS-I also occurs during S phase [48] and is linked to replication-coupled chromatin assembly. The S phase-specific increase in H4 K16ac levels is delayed in *cac1*Δ and *asf1*Δ mutants, and chromatin-associated H4 K16 is hypoacetylated in *cac1*Δ and *asf1*Δ mutants relative to wild-type [48–50]. Moreover, Sas2p and Cac1p interact in yeast two hybrid studies, and Asf1p or CAF-1 co-immunoprecipitate with SAS-I [51,52]. These data support a model where H4 K16ac deposition is regulated in a CAF-1 and Asf1p-dependent manner during replication-coupled chromatin assembly in S phase. Whether these assembly factors function independently or together to promote SAS-I-dependent H4 K16ac is unknown and how Rtt106p-mediated chromatin assembly influences H4 K16ac has not been explored previously.

We have examined the organization of replication-coupled chromatin assembly pathways within this network, how these pathways contribute to the deposition of appropriately modified histones during DNA replication and repair, and how this process promotes the formation of appropriate epigenetic states at individual loci. Here we show that CAF-1 interacts with Asf1p in live cells in a *RTT109*- and cell cycle-dependent manner, and a CAF-1-dependent chromatin assembly pathway restricts where silent chromatin can form by promoting H4 K16ac. In contrast, while interaction between Rtt106p and Asf1p similarly requires *RTT109*, H4 K16ac is independent of Rtt106p, and disruption of Rtt106p-dependent chromatin assembly as well as loss of Hat1p- or Hif1p-dependent pathways do not promote promiscuous silent chromatin formation. These and additional findings support a model in which this Rtt106p-dependent pathway is functionally separated from the CAF-1-dependent pathway at or downstream of Asf1p/Rtt109p. Furthermore, while Rtt101p promotes the Rtt106p-dependent pathway via ubiquitination of H3, Rtt101p also promotes a CAF-1-dependent pathway and H4 K16ac, but does so in a H3 ubiquitination-independent manner. Our findings imply processing of histones through different chromatin assembly pathways within this network during DNA replication will result in the deposition of histones with distinct modification patterns. These distinct patterns, in turn, can differentially influence the probability of forming new epigenetic states.

## Materials and methods

### Strain construction

Yeast strains used for this study are described in S1 Table and were generated using standard yeast genetic methods, including by genetic crosses, by plasmid shuffling to generate yeast strains expressing histone mutants (for plasmids see S2 Table) and by one-step gene conversion by homologous recombination to delete open reading frames (for primers see S3 Table) [53]. In general, at least two independent clones for each genotype were analyzed in experiments.

### Plasmid construction

Plasmids used for this study are listed in S2 Table. Plasmids expressing histone mutants were generated by site-directed mutagenesis using Phusion polymerase (NEB, cat# M0530S) and primers described in S3 Table as described previously [50].

### FLIM FRET

Fluorescence lifetime imaging microscopy and Förster resonance energy transfer, FLIM-FRET, was performed using scanning confocal time-resolved microscope systems; the Microtime 200 (Picoquant GmbH) (for all figures containing FLIM-FRET data except where noted in figure legend) as described previously [49,50], or an Alba (ISS, Champaign). For the Alba unit, a 488 nm picosecond pulsed laser with a 20 MHz repetition rate was used to excite GFP through a 60x apochromatic water immersion objective (NA = 1.2). Photons were collected by the same objective, reflected by a 560 nm dichroic filter (Chroma), then passed through a 50 μm pinhole to block off-focus photons, then further filtered with a band-pass filter (525/50 nm, Chroma) prior to detection via an avalanche photodiode (SPCM-AQRH-15, Excelitas) [54]. For both systems, the detected photons were stored in time-tagged time-resolved (TTTR) format to generate time-correlated single photon counting (TCSPC) histograms, and Fluorescence lifetimes plus FRET efficiency were calculated as described previously [49,50,54]. For lifetime analysis from Alba system, a threshold was used to exclude non-nucleus signal and a binning size of 7 pixels was used. The output image only contains lifetime data without intensity information. For each replicate, lifetime data was collected from ~15–20 cells with each morphology noted in figure legends and at least two independent replicates were performed for each pair condition. Representative lifetime images were collected during experiments.

### Cell fractionation

A 200 mL cell culture was grown to logarithmic phase (0.8–1 OD_600_/mL) for isolation of nuclei and subsequent chromatin fractionation as described previously [50].

### Protein blot analyses

Chromatin fractions were separated by electrophoresis on 15% SDS-PAGE gels, then transferred to PVDF membranes and processed as described previously [50] using anti-acetyl H4 K16 antibodies (Millipore, Cat# 07–329) (1:2000) for the primary antibody and HRP-conjugated anti-rabbit antibodies (GE Healthcare Life Sciences, Cat# NA934V) (1,10,000) as the secondary antibody. Membranes were stripped and reprobed with 1:6000 anti-H3 (Abcam, Cat# ab1791) (loading control) as outlined previously [50]. Blots were visualized using Luminata Crescendo Western HRP Substrate (Millipore) and imaged using ChemiDoc XRS+ System, then quantified using Image Lab Software 5.1. Data were calculated as follows: $\frac{H4K16ac_{mut}/H4K16ac_{WT}}{H3_{mut}/H3_{WT}}$, where mut = indicated strain, mean ± SD, *n* = 3. Statistical analyzes were conducted with the Wilcoxon rank-sum test using MSTAT v.6.5 (http://mcardle.oncology.wisc.edu/mstat).

Protein blot analysis of Sas5-YFPp levels in chromatin fractions was performed in a similar manner except blots were incubated overnight with anti-GFP (Genetex, Cat# GTX113617) (1:2500) at 4°C and then with HRP-conjugated anti-rabbit antibodies (GE Healthcare Life Sciences, Cat# NA934V) (1:10,000). Membranes were stripped, and re-probed with 1:5000 anti-PCNA antibodies [56,57], then incubated in HRP-conjugated anti-rabbit antibodies (GE Healthcare Life Sciences, Cat# NA934V) (1:10,000) at room temperature. Blots were then visualized and analyzed as above (see also [50]).

Protein blot analyses of H3 K56ac levels in chromatin fractions were performed similarly, except blots were initially probed with anti-H3 K56ac antibodies (Active Motif, Cat# 39281) (1:5000) overnight at 4°C, then with HRP-conjugated anti-rabbit antibodies (GE Healthcare Life Sciences, Cat# NA934V) (1:10,000), stripped and re-probed for H3 expression and visualized as above (see also [50]). Numerical data is provided in S4 Table.

### Patch mating assays

Cells were patched onto a YPD (1% Yeast extract, 2% Bacto Peptone, 2% D-Glucose) plate, or a minimal medium YM (6.7% Yeast Nitrogen Base without amino acids, 2% Glucose) plate with supplements, and grown overnight at 30°C. Cells were then replica plated onto a YPD plate as a control for growth and a *MAT****a*** *his4* tester lawn on a YM plate lacking supplements to test for silencing at *HMR* or *HMR****a***e**. The cells were then incubated at 30°C for one to two days (see [57]).

### Quantitative mating assays

Quantitative mating assays were conducted as described previously [57,58]. Statistical analyzes were conducted with the Wilcoxon rank-sum test using MSTAT v.6.5. Regression analyses with Bonferroni adjustments were also conducted to control for Type 1 error rates.

### Flow cytometry

One mL of logarithmically growing yeast were collected by centrifugation, resuspended in 70% ethanol (v/v with dH_2_O), and stored overnight at 4°C. Cells were washed 2X in FACS buffer (200 mM Tris-HCl, 20 mM EDTA, 0.001% NaN_3_), resuspended in 100 μL of 0.1% RNase in FACS buffer and incubated for two h at 37°C. Cells were washed one time with 1x PBS, then incubated in 100 μL propidium iodide solution (0.05 mg/mL propidium iodide in 1x PBS) for ≥ one h in the dark at 4°C. The sample volume was adjusted to one mL with 1x PBS. Samples were sonicated briefly (Branson Sonifier 450, VWR Scientific) prior to analysis by Flow Cytometry (BD Accuri C6, FlowJo software v.7.6.5).

### Colony color assays

Colony color assays were conducted as described previously (see [57]).

### Growth assays for synthetic interactions

Ten-fold serial dilution assays were conducted similar to as described previously [57,59]. Briefly yeast with indicated genotypes were grown logarithmically overnight in YPD or Complete Supplement Medium lacking tryptophan, CSM-TRP (6.7% Yeast Nitrogen Base without amino acids, 2% glucose, complete supplements lacking tryptophan), diluted to 1x10^4^ cells/μl, then 2.5 μl of 10-fold serial dilutions were spotted onto YPD medium under the conditions noted in the Figures.

### Chromatin immunoprecipitation

Yeast were grown logarithmically in YPD medium, then chromatin immunoprecipitation, ChIP, analyses were conducted using IgG (Diagenode, Cat. #C15410206), anti-histone H4 acetyl-Lys16 (Active Motif, Cat. #39167) or anti-H3 (Abcam, Cat. #ab1791) antibodies. Co-precipitated DNAs were analyzed by real-time PCR using an ABI Prism 7000 using oligonucleotides to monitor e** and ***a1*** as described previously [50,58–60]. Statistical analyses of ChIP data were conducted using the Wilcoxon rank-sum test with MSTAT v.6.5. Regression analyses with Bonferroni adjustments were also conducted to control for Type 1 error rates.

## Results

### Loss of CAF-1 and Asf1p, but not Rtt106p, Hif1p or Hat1p, promote silencing at HMRae**

**Fig 1:**
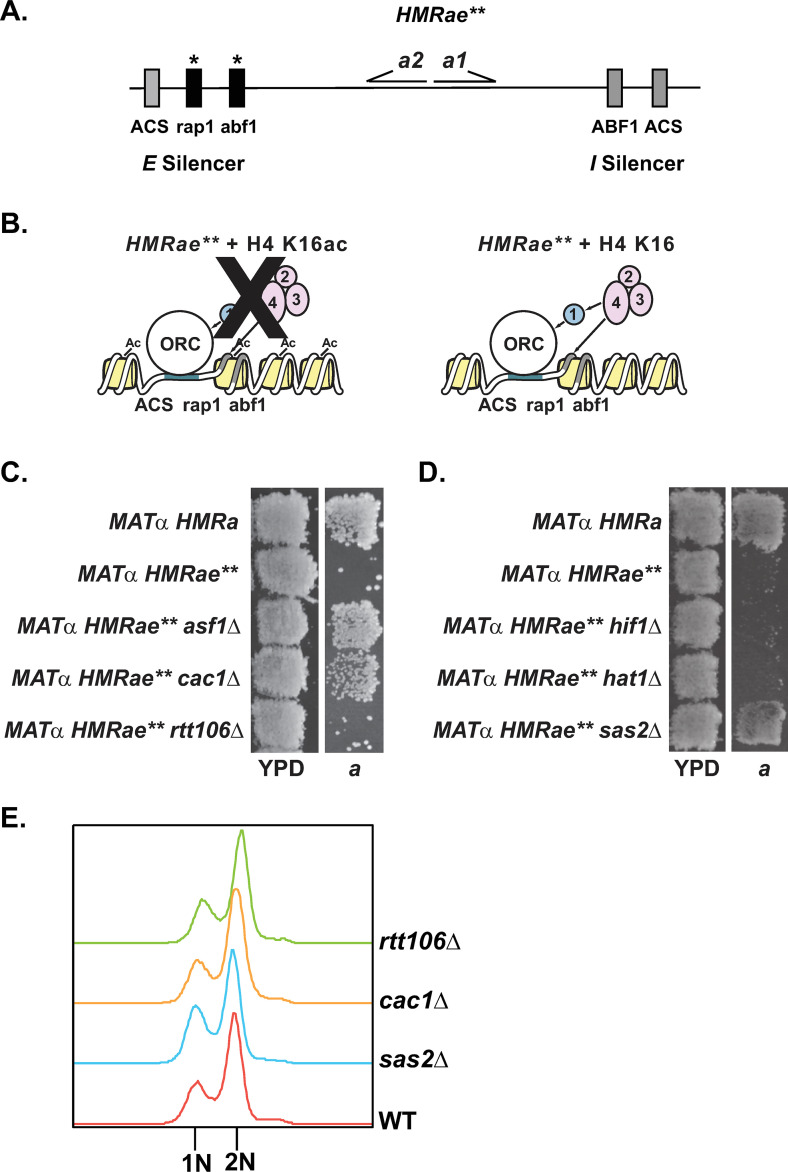
Loss of *CAC1* or *ASF1*, but not *RTT106*, restores silencing at HMRae**. **A)** Map of *HMR****a***e**. **B**) Mutants with defects in H4 K16ac promote Sir protein binding and restore silencing at *HMR****a***e**. 1, 2, 3, and 4 are Sir1p, Sir2p, Sir3p, and Sir4p, respectively. **C** and **D)** Patch Mating Assays. Cells with the indicated genotypes were grown on YPD at 30°C overnight, then were replica plated onto minimal medium (YM plate) with a *MAT****a*** lawn (JRY2726) and were grown at 30° for two days prior to imaging. Only cells that were silenced at *HMR****a***e** mated and grew as diploids on the *MAT****a*** plate. **E)** Flow Cytometry. Yeast with the indicated genotypes were grown logarithmically in YPD at 30°C prior to harvesting to assess cell cycle distribution by Flow Cytometry.

Strong evidence supports Asf1p functioning upstream of both CAF-1 and Rtt106p during DNA replication [4,19–25,28–30], and links between Asf1p and Hif1p during chromatin assembly have also been reported [40,41,42,61], but the distinct cellular functions of CAF-1, Rtt106p, and Hif1p have remained unclear. To evaluate their relationship and uncover distinct functions, we first examined silencing at the crippled *HMR****a***e** locus (Fig 1A). At *HMR****a***e**, the *E* silencer contains mutated Rap1p and Abf1p binding sites, which prevents Sir protein recruitment and silencing (Fig 1B), resulting in a non-mating phenotype for *MATα HMR****a***e** cells due to simultaneous expression of both ***a*** and α mating-type information [59,62]. However, certain second-site mutations leading to defects in H4 K16ac can restore silencing at this locus (Fig 1B) [52,59]. Similarly, silencing at *HMR****a***e** is restored in cells lacking *ASF1*, *RTT109*, or *CAC1* (Fig 1C) [50,52,57,59]. However, in contrast to *cac1*Δ and *asf1*Δ mutants, silencing at *HMR****a***e** was not restored in patch mating assays in either *rtt106*Δ or *hif1*Δ mutants (Fig 1C and 1D, see also [63]). Moreover, silencing was also not restored at *HMR****a***e** in cells lacking Hif1p’s interacting factor, Hat1p (Fig 1D) [42], or in H4 K5,12R mutants, which lack *HAT1*-dependent acetylation events associated with newly synthesized histone H4 [44,45,52]. Together, these results were consistent with the silencing phenotype at *HMR****a***e** having resulted from defects in a chromatin assembly pathway involving Asf1p and CAF-1, rather than Rtt106p or Hif1p. These results also supported a model in which histones H3/H4 could enter the pathway involving Asf1p and CAF-1 via a HAT-B or NuB4-independent process (see Discussion).

### Chromatin-associated H4 K16ac does not require *RTT106*, but is defective in *rtt109*Δ mutants

The differing ability of *cac1*Δ and *rtt106*Δ mutants to restore silencing at *HMR****a***e** raised the possibility that important and distinct functions of Rtt106p and CAF-1 are to assemble differentially modified chromatin during DNA replication. Like in *asf1*Δ and *cac1*Δ mutants, loss of any one of the subunits of the H4 K16-specific acetyltransferase complex SAS-I (encoded by *SAS2*, *SAS4*, or *SAS5*) restores silencing to *HMR****a***e** (Fig 1D) [52,64–66] as does a catalytically inactive mutant of *SAS2* [52]. Moreover, cells lacking *SAS2*, *CAC1* or *ASF1* have decreased levels of chromatin-associated H4 K16ac (Table 1, [49,50]). As loss of *CAC1*, *ASF1*, or *RTT109* [50, 52, 57, 59], but not *RTT106* (Fig 1C), restored silencing at *HMR****a***e**, we predicted that, like *asf1*Δ and *cac1*Δ mutants, *rtt109*Δ, but not *rtt106*Δ, mutants would exhibit defects in H4 K16ac. To test this prediction, we analyzed H4 K16ac levels in chromatin fractions isolated from logarithmically growing *rtt109*Δ, *rtt106*Δ, *cac1*Δ, or *sas2*Δ mutants relative to wild-type cells. *rtt106*Δ mutants had similar levels of H4 K16ac as in wild-type cells. In contrast, reduced levels of H4 K16ac were observed in *rtt109*Δ mutants, similar to *cac1*Δ and *sas2*Δ mutants (Table 1 and S1 Fig, see also [49]). As acetylation of H4 K16 is cell cycle-regulated [48], one explanation for these observations could have been that *rtt106*Δ mutants were enriched in S phase cells, whereas the *cac1*Δ mutants had accumulated outside of S phase. However, when we monitored the cell cycle distribution of logarithmic cultures of each mutant as well as wild-type by flow cytometry, their cell cycle distributions were similar to wild-type (Fig 1E). Together, these results were consistent with H4 K16 hypoacetylation at *HMR****a***e** promoting restoration of silencing (see also [52,59]) and deposition of H4 K16ac occurring through an Asf1p/Rtt109p/CAF-1-mediated pathway that functions independently of Rtt106p.

**Table 1:** *rtt109* mutants have defects in chromatin-associated H4 K16ac.

Genotype	Relative Levels of H4 K16ac^1^
Wild-type	1
*sas2*Δ	0.42 ± 0.056^2^
*rtt106*Δ	0.91 ± 0.24
*cac1*Δ	0.61 ± 0.22^2^
*rtt109*Δ	0.37 ± 0.13^2^

^1^The relative level of chromatin-associated H4 K16ac in each strain was determined by quantitative protein blot analyses, was normalized to H3, and expressed relative to that observed in wild-type cells, which was set to 1 (see Materials and Methods). Avg. ± St. Dev., n = 3. Numerical data is in S4 Table.

^2^P = 0.03, Wilcoxon rank-sum test. (Representative immunoblot shown in S1 Fig).

### Sas5p association with chromatin in the absence of chromatin assembly factors

We have previously demonstrated SAS-I, a complex responsible for H4 K16ac, interacts with PCNA, and this interaction is disrupted when cells express *pol30* mutants with defects primarily in Asf1p- or CAF-1-dependent chromatin assembly pathways [49]. This data, along with the observation that chromatin-associated H4 K16ac levels are also decreased in *pol30*, *cac1*Δ, and *asf1*Δ mutants, but not in *rtt106*Δ mutants (Table 1 and S1 Fig) [49], raised the possibility that recruitment of SAS-I to chromatin was dependent on one of these factors. Therefore, we analyzed Sas5p levels in chromatin fractions isolated from wild-type, *cac1*Δ, *rtt106*Δ, *asf1*Δ and *rtt109*Δ mutants by immunoblotting. However, the level of chromatin-associated Sas5p in *rtt106*Δ, *cac1*Δ or *asf1*Δ mutants remained similar to wild-type, but was mildly reduced in *rtt109*Δ mutants (Table 2 and S2 Fig). Together, these observations are consistent with SAS-I being recruited to chromatin through multiple mechanisms, but how this occurs awaits further study.

**Table 2:** Sas5p associates with chromatin in cells lacking *ASF1* or *CAC1*.

Genotype	Relative Levels of Sas5-YFP^1^
*SAS5-YFP*	1
*SAS5-YFP cac1*Δ	1.1 ± 0.29
*SAS5-YFP rtt106*Δ	0.90 ± 0.27
*SAS5-YFP asf1*Δ	0.99 ± 0.044
*SAS5-YFP rtt109*Δ	0.75 ± 0.12^2^

^1^The relative level of chromatin-associated Sas5-YFPp in each strain was determined by quantitative protein blot analyses, was normalized to H3, and expressed relative to that observed in wild-type cells, which was set to 1 (see Materials and Methods). Avg. ± St. Dev., n = 4. Numerical data is in S4 Table.

^2^P = 0.03; Wilcoxon Rank-Sum test. (Representative immunoblot shown in S2 Fig).

### Asf1p interacts with Cac1p and Rtt106p in a *RTT109-*dependent manner *in vivo*

**Fig 2:**
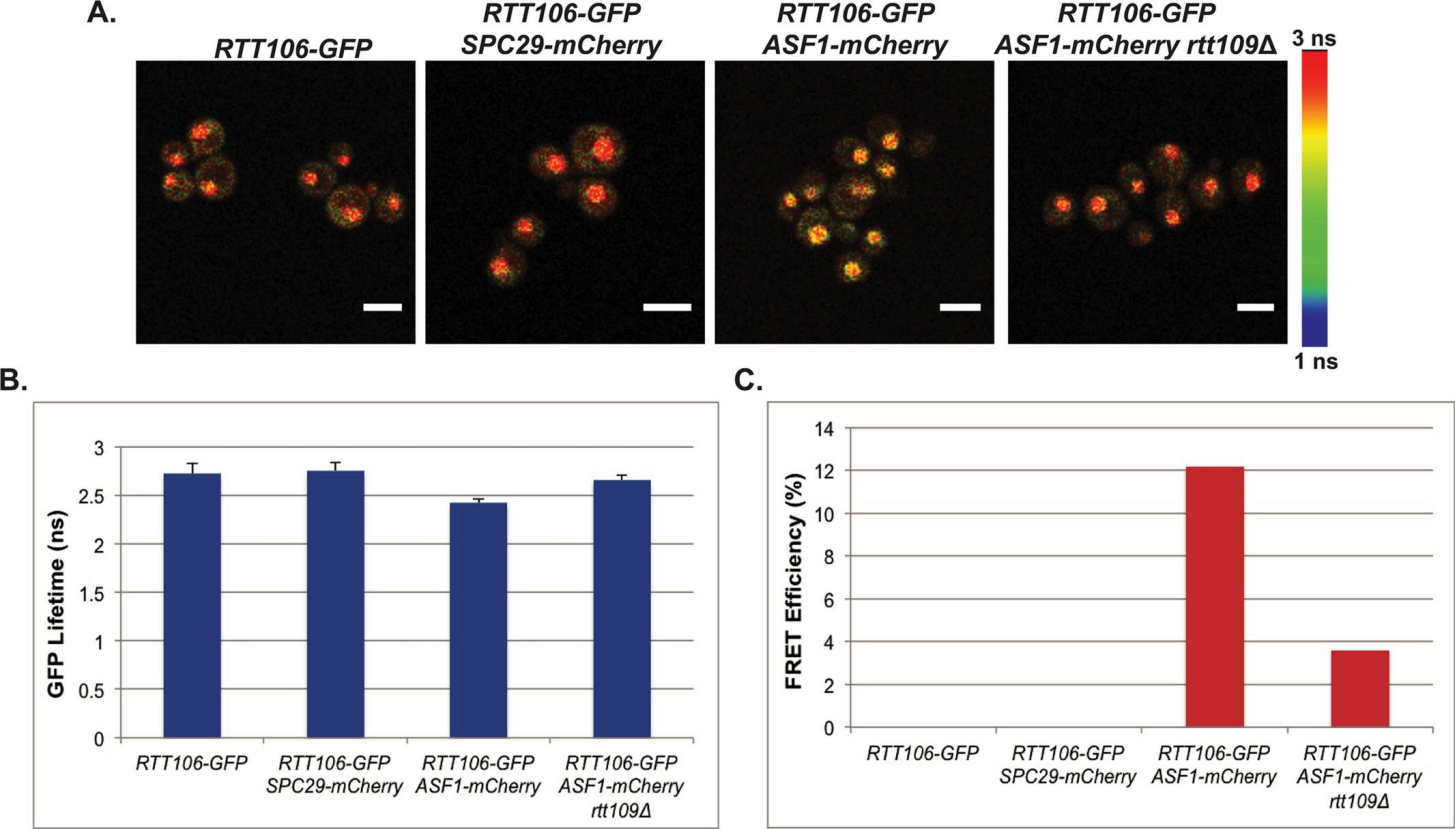
Rtt106p and Asf1p interact in a *RTT109*-dependent manner *in vivo*. **A)** Confocal fluorescence lifetime images of GFP in small-budded live cells expressing the fluorescently-tagged proteins as indicated. White scale bars are equivalent to 5μm. FLIM scale bar: 1 nanosecond, blue; 3 nanoseconds, red. **B)** The average lifetime of GFP in indicated strains. Error bars represent the standard deviation of ten FLIM measurements taken for each genotype. **C)** FRET efficiency of indicated strains.

We next explored protein-protein interactions amongst these factors in live cells to examine their relationship during chromatin assembly. As the interactions between H3 and Cac1p or Rtt106p are promoted by H3 K56ac [4,21], we predicted that previously observed interactions between Asf1p and Cac1p or Rtt106p may require *RTT109* [35,67]. To test this, we first assessed interactions between Asf1p and Rtt106p by measuring the fluorescent lifetime of GFP in live cells expressing either Asf1-GFPp alone, or Asf1-GFPp plus Rtt106-mCherryp, or negative control Spc29-mCherryp by FLIM-FRET. In FLIM-FRET, if a FRET interaction occurs between the donor (GFP)-tagged protein and the acceptor (mCherry)-tagged protein, the lifetime of the donor in the excited state will decrease relative to the cells expressing only the donor-tagged protein. In this analysis, we evaluated interactions between Rtt106-GFPp and Asf1-mCherry in small budded cells as the association of H3 and Rtt106p is cell cycle-dependent and peaks in S phase [4]. A decrease in the lifetime of GFP was observed in small-budded cells with a single nucleus expressing both Rtt106-GFPp and Asf1-mCherryp relative to cells expressing Rtt106-GFPp alone or Rtt106-GFPp plus the Spc29-mCherry control, indicating that Asf1p interacted with Rtt106p *in vivo* [Fig 2A and 2B, see also [4,34]]. The FRET efficiency between Rtt106-GFPp and Asf1-mCherryp was calculated from the lifetimes obtained from the TCSPC decay histograms that were fitted with a double exponential function, and the FRET efficiency of Rtt106-GFPp with Asf1-mCherryp was 12% (Fig 2C). Loss of *RTT109* in Rtt106-GFPp Asf1-mCherryp cells resulted in a lifetime of GFP that was similar to that of cells expressing Rtt106-GFPp alone (Fig 2A and 2B) and FRET interactions were lost in the *rtt109*Δ mutants (Fig 2C), indicating that the interaction between Rtt106p and Asf1p was dependent on *RTT109*.

**Fig 3:**
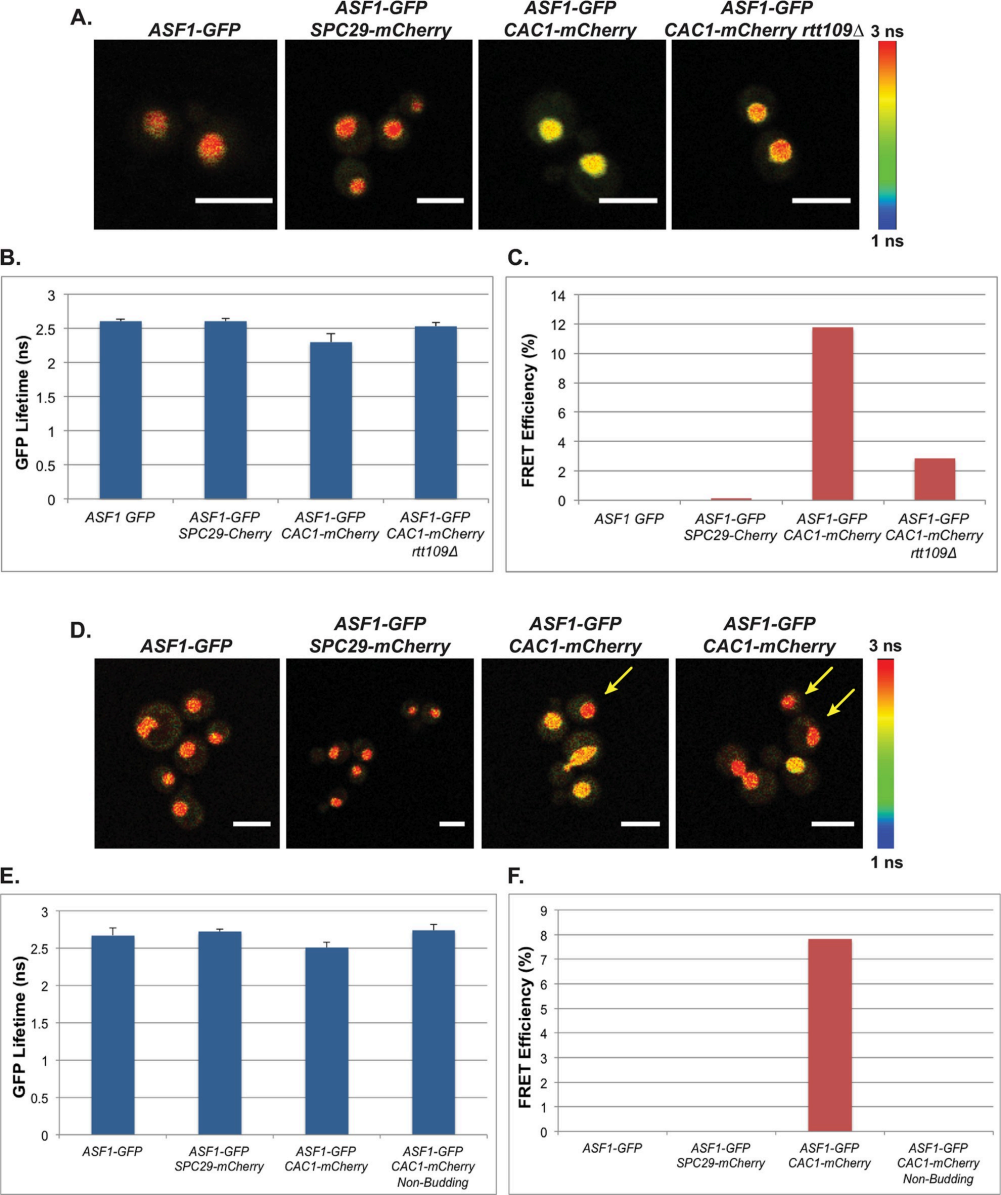
Cac1p and Asf1p interact in a cell cycle- and *RTT109*-dependent manner *in vivo*. **(A-C)** Interaction between Cac1p and Asf1p is *RTT109*-dependent. **A)** Confocal fluorescence lifetime images of GFP in small-budded live cells expressing the fluorescently-tagged proteins as indicated. **B)** The average lifetime of GFP in indicated strains. **C)** FRET efficiency of indicated strains. **(D-F)** Interaction between Cac1p and Asf1p is cell cycle-dependent. **D)** Confocal fluorescence lifetime images of GFP in unbudded and budded live cells expressing the fluorescently-tagged proteins as indicated. Left panels arrows = unbudded cells. **E)** The average lifetime of GFP in indicated strains. **F)** FRET efficiency of indicated strains. Error bars represent the standard deviation of ten FLIM measurements taken for each genotype. White scale bars are equivalent to 5μm. FLIM scale bar: 1 nanosecond, blue; 3 nanoseconds, red.

To assess interactions between Asf1p and Cac1p, we measured the lifetime of GFP in live cells expressing either Asf1-GFPp alone, or Asf1-GFPp plus Cac1-mCherryp or negative control Spc29-mCherryp by FLIM-FRET. In this analysis, the lifetime of GFP in small-budded cells with a single nucleus expressing both Asf1-GFPp and Cac1-mCherryp decreased relative to that observed in cells expressing Asf1-GFPp alone or the Spc29-mCherryp control (Fig 3A and 3B; see also Fig 3D and 3E). To quantitate these protein-protein interactions, FRET efficiency between Asf1-GFPp and Cac1-mCherryp was calculated as above. The FRET efficiency of Asf1-GFPp with Cac1-mCherryp in two independent analyses was 12 or 8%, whereas no interaction was observed in cells expressing Asf1-GFPp and the control Spc9-mCherryp (Fig 3C–3F, respectively). In contrast, interaction between Asf1-GFPp and Cac1-mCherryp was lost in unbudded cells (Fig 3D–3F), indicating Asf1p and Cac1p interacted in a cell cycle-dependent manner *in vivo* [4,22]. Loss of this interaction could also be observed in occasional cells containing two nuclei (e.g. Fig 3D, right panel), but whether the loss of FRET was synchronous as cells passed through mitosis was not determined. When *RTT109* was deleted in these cells, the lifetime of GFP did not change in small budded cells containing a single nucleus relative to cells expressing Asf1-GFPp alone, and FRET interactions were lost, indicating that the interaction between Asf1p and Cac1p *in vivo* was *RTT109-*dependent (Fig 3A–3C). In support of this interaction being linked to DNA replication-coupled chromatin assembly, Asf1p interacted with PCNA in similar FLIM-FRET analyses (S3 Fig), and we have previously demonstrated that SAS-I and Rtt109p similarly interact with wild-type PCNA, but not with PCNA (pol30-6p) mutants that have defects in *ASF1*-dependent pathways, or with PCNA (pol30-8p) mutants that have defects in CAF-1-dependent pathways [49]. Collectively, these data imply that not only does Rtt109p*/*H3 K56ac play a role in promoting transfer of histones from Asf1p to CAF-1 or to Rtt106p, but also that *RTT109* is required for association of Asf1p with CAF-1 or Rtt106p *in vivo*.

### Overexpression of *SAS2* disrupts silencing at HMRae** in *rtt109*Δ mutants

**Fig 4:**
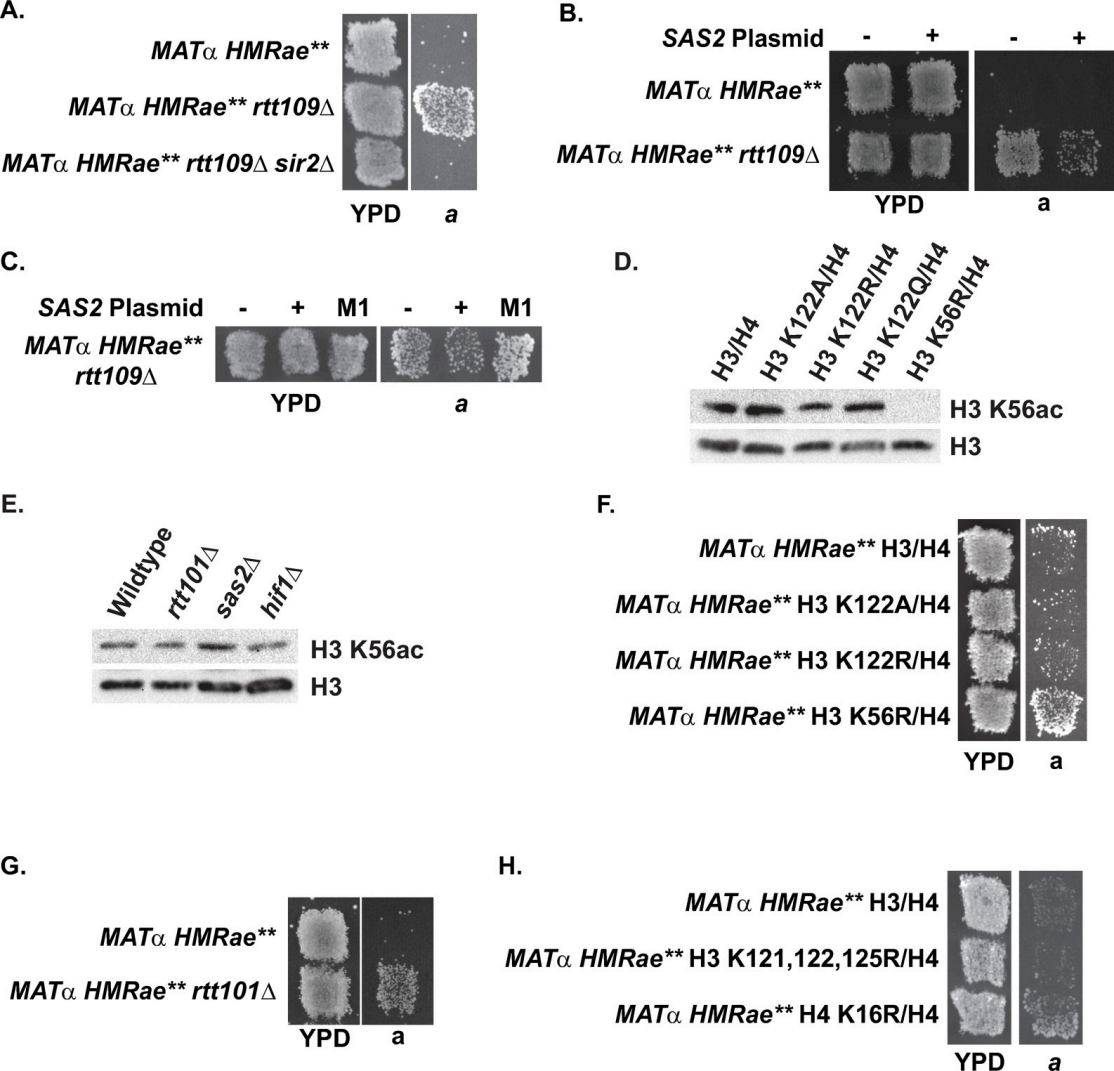
Effects of histone modifications associated with replication-coupled chromatin assembly on silencing at HMRae**. **A)** Loss of *RTT109* restored silencing at *HMR****a***e** in a *SIR2*-dependent manner. **B)** Overexpression of *SAS2* in *rtt109*Δ mutants disrupted silencing at *HMR****a***e**. **C)** The catalytic activity of Sas2p was required to disrupt silencing at *HMR****a***e** in *rtt109*Δ mutants. **D)** Acetylation of H3 K56 did not require H3 K122ub. **E)** Acetylation of H3 K56 did not require *RTT101*, *SAS2* or *HIF1*. **F)** Loss of acetylation at H3 K56, but not of ubiquitination at H3 K122 restored silencing at *HMR****a***e** (see also Table 3). **G-H)** Loss of *RTT101* **(G)**, but not of ubiquitination at H3 K121, K122 plus K125 **(H)** restored silencing at *HMR****a***e**. Cells with the indicated genotypes were analyzed as in Fig 1.

Combined, the observations that loss of H4 K16ac [49,50,52,59], *ASF1* (Fig 1C, see also [52, 59]) or *CAC1* (Fig 1C, see also [50,57]) restore silencing at *HMR****a***e**, and that *RTT109* was required for interactions between Asf1p and Cac1p *in vivo* (Fig 3A–3C), implied that Rtt109p and H3 K56ac functioned upstream of H4 K16ac. Consistent with this model, chromatin-associated H4 K16ac levels were decreased in *rtt109*Δ mutants relative to wild-type cells (Table 1 and S1 Fig), and loss of *RTT109* restored silencing at *HMR****a***e** in a *SIR2*-dependent manner in patch mating assays (Fig 4A, see also [59]). Moreover, silencing at *HMR****a***e** in *rtt109*Δ mutants was disrupted upon overexpression of *SAS2* (Fig 4B), but not catalytically inactive sas2-M1p (Fig 4C) [51,68], indicating suppression required the catalytic activity of Sas2p. To determine if the observed *SAS2*-mediated suppression was stable, several colonies from single parental clones were expanded individually and analyzed by patch mating. These subclones varied in their ability to mate (S4 Fig). Thus, overexpression of *SAS2* disrupted silencing at *HMR****a***e** mediated by loss of *RTT109*, but in a variegated manner, consistent with overexpression of *SAS2* having negatively influenced the probability of establishing the silenced state.

### Loss of *RTT101*, but not H3 K122A or H3 K122R, restores silencing at HMRae**

Recently, ubiquitination of H3 has been proposed to promote the transfer of histones from Asf1p to Rtt106p based on observations that in cells with defects in H3 ubiquitination mediated by the cullin Rtt101p, H3 co-precipitation with Asf1p is increased, and co-precipitation of H3 with Rtt106p is reduced, whereas co-precipitation between Cac2p and H3 is unaffected [33]. Our new observations presented the opportunity to test this prediction as if loss of ubiquitination of H3 disrupted a *RTT106*-mediated chromatin assembly pathway, then H3 mutants with defects in ubiquitination would be unable to silence *HMR****a***e**, as was the case for *rtt106*Δ mutants. Therefore, we analyzed H3 K122A and H3 K122R mutants as K122 is the primary residue on H3 that is ubiquitinated in a *RTT101*-dependent manner [33]. Ubiquitination of H3 K122 (Fig 4D) and *RTT101* (Fig 4E) were not required for acetylation on K56 of chromatin-associated histone H3 (see also [33,69]). In contrast to H3 K56R mutants, and similar to *rtt106*Δ mutants, H3 K122A and H3 K122R mutants did not restore silencing at *HMR****a***e** (Fig 4F and Table 3 [59]). Surprisingly, however, silencing was restored at *HMR****a***e** cells lacking *RTT101* (Fig 4G). One possible explanation for the differing phenotypes between H3 K122 and *rtt101*Δ mutants was that ubiquitination of a different substrate of Rtt101p may have influenced the probability of establishing silencing at *HMR****a***e**. As K121 and K125 of histone H3 can also be ubiquitinated in a *RTT101*-dependent manner, we tested silencing at *HMR****a***e** in H3 K121, 122, 125R mutants (Fig 4H), but silencing was not rescued in this genetic background either, in contrast to H4 K16R mutants. Together, these results were consistent with Rtt101p functioning in a histone H3 ubiquitination-independent process that normally prevents silent chromatin from forming at “off target sites” such as that illustrated by *HMR****a***e** (see below).

**Table 3:** Loss of H3 K122 ubiquitination does not restore silencing at HMRae**.

Strain	Relative Efficiency of Mating^1^
H3/H4	1
H3 K122A/H4	0.65 ± 0.29^2^
H3 K122R/H4	1.0 ± 0.45
H3 K56R/H4	8.5 ± 3.7^2^

^1^The efficiency of mating of *MAT*α *HMR****a***e** cells expressing wild-type H3-H4 to tester strain JRY2726 (*MAT****a***) was determined relative to their plating efficiency (1.2 ± 0.025%, n = 4), and set to 1. The mating efficiency of each strain relative to *MAT*α *HMR****a***e** cells with wild-type H3 H4 was determined as outlined in Materials and Methods. Avg. ± St. Dev.; n = 3 or 4. Numerical data is in S4 Table.

^2^P = 0.024; Wilcoxon Rank-Sum test.

### H3 K122 mutants and *cac1*Δ, but not *rtt106*Δ, display synthetic silencing defects

To further explore the relationship between chromatin assembly pathways, *RTT101-*dependent ubiquitination of H3, and additional aspects of silencing, we analyzed synthetic effects on silencing at *HMR* between H3 K122 mutants and *cac1*Δ, *rtt106*Δ, *hif1*Δ, *hat1*Δ, *rtt109*Δ, or *asf1*Δ mutants using the *HMR*::*ADE2* reporter, which contains wild-type silencers flanking the reporter gene (S5 Fig). Multiple H3 K122 mutants were chosen instead of *RTT101* in these analyses for specificity, as Rtt101p interacts with multiple proteins that function in numerous processes in chromatin biology ranging from the initiation of DNA replication to DNA repair to sister chromatid cohesion to silencing (e.g. [70–74]), and other post-translational modifications to this H3 residue, in addition to ubiquitination, have been reported in other organisms [77–79]. In contrast to *HMR****a***e**, which exclusively assesses changes to the locus that promote the silent state of *HMR****a***e** during G1 phase in individual cells via measuring G1 phase-specific mating events, the *HMR*::*ADE2* reporter (S5A Fig) can instead be used to monitor defects in silencing at a locus containing wild-type *E* and *I* Silencers as well as defects in the longer-term stability, or maintenance, of the silenced state across cell generations. In wild-type cells, *HMR*::*ADE2* is silenced and colonies are red in color. However, if mutant yeast have defects in silencing, their colonies may be pink, sectored, or white, depending on the nature and severity of the silencing defect. In our analyses, wild-type cells expressing H3 grew as red colonies (S5B–S5E Fig), whereas expression of H3 K122R in otherwise wild-type cells resulted in colonies becoming light pink, which indicated a defect in maintaining or inheriting silent chromatin had occurred in the presence of a positive charge plus the absence of ubiquitination at this residue (S5B Fig).

Expression of H3 K122Q (S5C Fig) or H3 K122A (S5D and S5E Fig) in an otherwise wild-type cell also resulted in pink colonies. *cac1*Δ mutants grew as sectored colonies (see also [49,80]), but when combined with H3 K122A or H3 K122R, the colonies were white (S5B, S5D and S5E Fig). This negative synthetic interaction between *cac1*Δ and H3 K122 mutants demonstrated Cac1p and H3 K122ub operate in separate pathways with regards to silencing *HMR*::*ADE2*. However, expression of H3 K122Q in *cac1*Δ mutants did not lead to more severe silencing defects relative to single mutants (S5C Fig), indicating that ubiquitination on H3 *per se* was not critical for silencing, rather the charge state present at this residue, or at least the surface of H3 at this location, was. Like *cac1*Δ mutants, *rtt106*Δ mutants also exhibited a silencing defect, but expression of the H3 K122R or H3 K122A in *rtt106*Δ mutants did not result in synthetic defects in silencing relative to single mutants (S5B, S5D and S5E Fig), which is consistent with Rtt106p and H3 K122ub operating in the same pathway with regards to silencing *HMR*:: *ADE2*. However, in contrast to *cac1*Δ mutants, expression of H3 K122Q in *rtt106*Δ mutants lead to growth defects relative to single mutants (S5C Fig), precluding reliable assessment of their silencing phenotype under the conditions tested. Growth defects, often severe, were also observed when *asf1*Δ or *rtt109*Δ mutants were combined with H3 K122 mutants, with silencing being lost in the *rtt09*Δ H3 K122Q mutants (S5B–S5E Fig, described in more detail below).

Although loss of *HIF1* or *HAT1* could not restore silencing at *HMR****a***e** (in contrast to *asf1*Δ or *cac1*Δ mutants; Fig 1), physical interactions between Asf1/H3-H4 and HAT-B or NuB4 have been reported [42,47,81]. H4 K5, 12ac copurifies with Asf1p [34], and H4 that copurifies with Cac2p can contain acetylated K5 and K12, both with and without H4 K16ac [82] (see also [83]). Such observations are consistent with HAT-B and/or NuB4 functioning, at least in part, upstream of CAF-1, and potentially Rtt106p as well, during replication-coupled chromatin assembly. Therefore, we also evaluated silencing in H3 K122A/R/Q mutants combined with *hif1*Δ or *hat1*Δ mutants. In contrast to loss of *CAC1*, and more similar to loss of *RTT106*, loss of *HIF1*or *HAT1* in H3 K122A mutants did not lead to more severe silencing defects than those observed for the individual H3 K122A mutants (S5D and S5E Fig). These results were consistent with HAT-B or NuB4 functioning in the same pathway as H3 K122ub with respect to silencing. However, as more severe silencing defects were observed e.g. in H3 K122R and *hif1*Δ H3 K122R mutants (S5B Fig), this surface of H3 appears to have at least one additional function important for silencing. Combined, these results indicated H3 K122 mutants with defects in silencing were differentially sensitive to perturbations to replication-coupled chromatin assembly pathways.

### H3 ubiquitin-deficient mutants display synthetic growth defects with mutants defective in modifying histones or assembling chromatin

To better characterize the genetic relationships between H3 ubiquitination mutants and factors associated with modifying histones and/or replication-coupled chromatin assembly, *cac1*Δ, *rtt106*Δ, *hif1*Δ, *hat1*Δ, *rtt109*Δ, *asf1*Δ, *sas2*Δ or *dot1*Δ mutants expressing H3 K122A/R/Q or wild-type H3/H4 were tested for synthetic interactions that could be revealed by growth under different temperatures (S6 and S7 Figs). Each single H3 K122 mutant in otherwise wild-type cells exhibited mild growth defects in serial dilution growth assays, but H3 K122Q mutants exhibited more severe growth defects at 23°C relative to cells expressing wild-type H3 (S6 and S7 Figs; see also [84]). However, *cac1*Δ mutants expressing H3 K122R or H3 K122A had more severe growth defects relative to single mutants or wild-type at all temperatures tested, 23°C, 30°C, and 35°C, with largest growth defects observed at both 23°C and 35°C (S6A and S7 Figs). These defects could be suppressed by exogenous expression of *CAC1* or *cac1* mutants mimicking unphosphorylated or phosphorylated forms of Cac1p (S8 and S9 Figs) [50], implying that the observed synthetic defects were not related to pathways associated with phosphorylation of Cac1p. *cac1*Δ H3 K122Q mutants grew more efficiently relative to wild-type than *cac1*Δ H3 K122R or H3 K122A mutants at all temperatures tested (S6 and S7 Figs), and *cac1*Δ H3 K122Q mutants grew with similar efficiency as single H3 K122Q mutants (S6A Fig), implying the charge of this residue affected whether the mutant compromised a pathway separate from *CAC1*. In contrast to *cac1*Δ H3 K122Q mutants, *rtt106*Δ H3 K122Q mutants exhibited a synthetic defect in growth at all temperatures relative to single mutants (S6A Fig), whereas expression of H3 K122A in *rtt106*Δ mutants did not adversely affect growth rate or viability (S7 Fig), and *rtt106*Δ H3 K122R mutants exhibited a slight growth defect at 23°C and 35°C, but not at 30°C relative to single mutants (S6A Fig). Together these results are consistent with the charge state of H3 K122, in addition to the ubiquitination state, differentially contributing to synthetic growth defect upon loss of *CAC1* or *RTT106*.

We also explored if the genetic interactions between H3 K122R/Q observed above could be linked to defects in Sas2p-dependent acetylation of H4 K16 [49], Dot1p-dependent methylation of H3 K79 [85,86], or Rtt109p-dependent acetylation of H3 K56. H3/H4 co-purifying with CAF1 are acetylated on H4 K16 [82], methylated on H3 K79 [82], and acetylated on H3 K56 [82,87]. Loss of *SAS2* or *RTT109* or expression of H4 K16R or H3 K56R, but not *DOT1* or expression of H3 K79R, restores silencing at *HMR****a***e** (Fig 1D and [52,59,66,88]). However, the single H3 K122R or H3K122Q mutants and *sas2*Δ or *dot1*Δ double mutants grew similarly at each temperature (S6B and S6C Fig), supporting a model in which the observed negative genetics interactions between K122 mutants and *cac1* mutants did not require Sas2p-mediated acetylation of H4 K16 or Dot1p-mediated methylation of H3 K79. In contrast to *sas2*Δ and *dot1*Δ mutants, expression of H3 K122A or H3 K122Q in *rtt109*Δ mutants resulted in severe growth defects compared to either single mutants at all temperatures and conditions tested (S6B and S7 Figs; see also S5 Fig). Similar results were observed in *asf1*Δ H3 K122Q mutants (S6B Fig; see also S5 Fig). *asf1*Δ H3 K122A mutants could not be tested as this combination was lethal during plasmid shuffling. *asf1*Δ H3 K122R and *rtt109*Δ H3 K122R mutants also exhibited synthetic growth defects at 35°C (S6B Fig). Thus, in the absence of *ASF1* or *RTT109*, the status of residue 122 on H3 became critical for growth.

**Fig 5:**
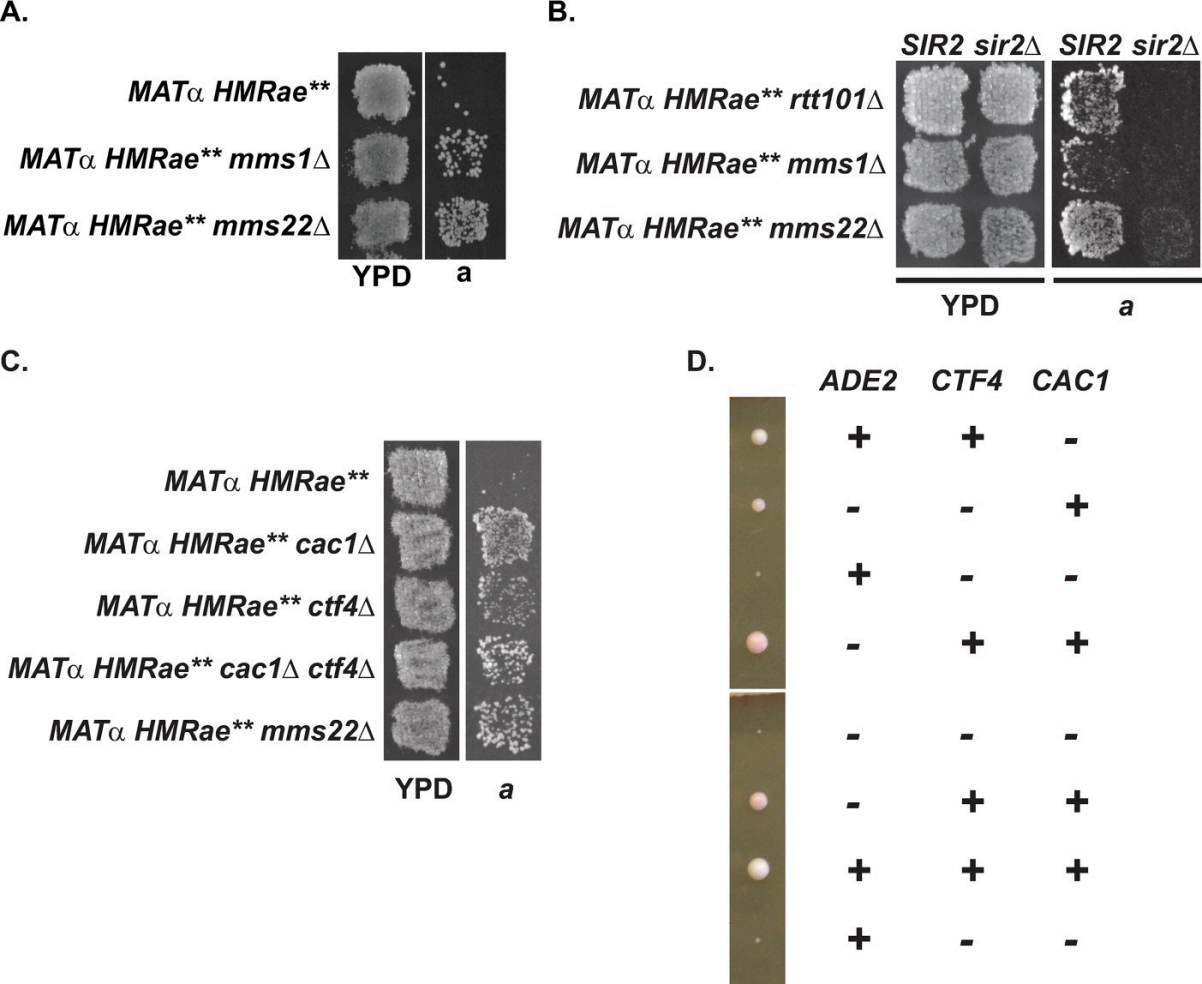
Defects in factors associated with sister chromatid cohesion restore silencing at HMRae**. **A-B)** Restoration of silencing at *HMR****a***e** in *mms1*Δ, *mms22*Δ **(A)**, and *rtt101*Δ **(B)** mutants required *SIR2*. **C)** Loss of *CTF4* restored silencing at *HMR****a***e**. For Patch Mating assays, cells with the indicated genotypes were analyzed as in Fig 1**D)** Negative synthetic interaction between *ctf4*Δ and *cac1*Δ mutants. Tetrads from diploid strain *ade2-1*/*ADE2 cac1*Δ/*CAC1 CTF4***/***ctf4*Δ were dissected onto YPD plates and incubated three days at 30°C prior to imaging.

**Fig 6:**
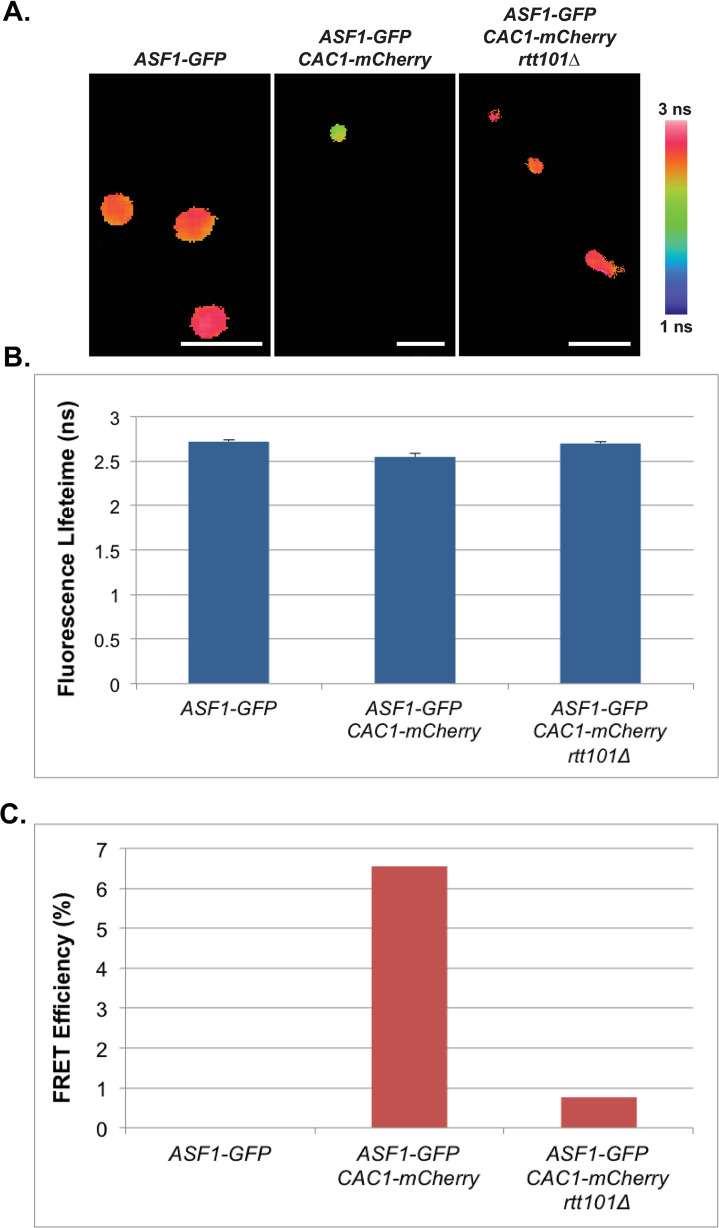
Cac1p and Asf1p interact in a *RTT101-*dependent manner *in vivo*. **A)** Confocal fluorescence lifetime images of GFP in small-budded live cells expressing the fluorescently-tagged proteins as indicated. White scale bars are equivalent to 5μm. FLIM scale bar: 1 nanosecond, blue; 3 nanoseconds, pink. **B)** The average lifetime of GFP in indicated strains. Error bars represent the standard deviation of ten FLIM measurements taken of each sample. **C)** FRET efficiency of indicated strains. Data were collected on an Alba (ISS, Champaign).

When we evaluated genetic interactions between H3 K122A/R/Q and *hif1*Δ or *hat1*Δ mutants for temperature sensitivity, *hif1*Δ H3 K122A/R/Q mutants grew with similar efficiency as H3 K122A/R/Q single mutants (Figs 5 and 6A and S7 Fig), but *hat1*Δ H3 K122Q mutants exhibited growth defects relative to either single mutant at 30°C and 35°C (S6C Fig, see also S5C Fig). Also, *hat1*Δ H3 K122R mutants were also sensitive to 35°C (S6C Fig), whereas *hat1*Δ H3 K122A did not exhibit sensitivity compared to single mutants (S7 Fig, see also S5 Fig). When considered together with the data above, these results further supported a model in which HAT-B or NuB4 were not the only upstream sources of histones H3/H4 for Asf1p.

Finally, to assess how loss of all *RTT101*-dependent ubiquitination events on H3 influenced growth in the absence of factors linked to replication coupled chromatin assembly, we expanded the synthetic interaction analyses in the context of H3 K122,125R (S10 Fig) and H3 K121,122,125R mutants (S11 Fig). H3 K122,125R exhibited mild growth defects relative to wild-type at 23°C, and these growth defects were enhanced at various temperatures in the absence of *CAC1*, *RTT106*, or *HAT1* (S10 Fig). H3 K121,122,125R mutants exhibited mild growth defects relative to yeast expressing wild-type H3 at 30°C, but had more severe growth defects at both 23°C and 35°C (S11 Fig). These defects were enhanced in the absence of *CAC1*, *RTT106*, *ASF1*, *RTT109* or *HAT1*, but not *HIF1*, *SAS2* or *DOT1* (S11 Fig). Thus, the negative genetic interactions with the H3 ubiquitination mutants followed similar trends, except more severe defects were observed for H3 K121,122,125R mutants lacking *RTT106* or *HAT1*. Combined, these data support a model in which ubiquitination of H3 becomes critical in the absence of *ASF1* and *RTT109-*dependent chromatin assembly. Determining whether these synthetic interactions reflect the existence of an additional, H3 K122ub-dependent, chromatin assembly pathway that functions in parallel to an Asf1p-Rtt19p-dependent pathway during DNA replication or reflects interactions related to *ASF1*-dependent functions during transcription [89] awaits further studies. These data also indicate the charge status of H3 K122 differentially contributes to viability, depending on which pathways linked to replication-coupled chromatin assembly remained intact.

### H3 ubiquitin-deficient mutants are hypersensitive to DNA damage

As several factors involved in replication-coupled chromatin assembly in addition Rtt101p contribute to cellular responses to DNA damage (e.g. [59,90,91]), we extended the above synthetic interaction analyses to include growth at 30°C upon exposure to UV light (S6, S7, S8A, S9A, S10 and S11 Figs) and other DNA damaging agents (see below) to better understand the functional organization of this chromatin assembly network. We observed H3 K122 mutants were not hypersensitive to exposure to UV relative to wild-type under the conditions tested (S6, S7, S8A and S9A Figs), but expression of H3 K122R/Q/A, or H3 K122,125R mutants in cells lacking *CAC1* resulted in increased sensitivity to UV light relative to the single mutants (S6A, S7, S8A, S9A and S10 Figs). This sensitivity of H3 K122R *cac1*Δ mutants, but not H3 K122Q *cac1*Δ mutants, was suppressed by exogenous expression of *CAC1* or *cac1* phosphorylation mutants (S8A and S9A Figs). H3 K122R, H3 K122Q, H3 K122,125R, and H3 K121,122,125R *rtt106*Δ double mutants, but not H3 K122A *rtt106*Δ mutants, also exhibited increased sensitivity to UV compared to the single mutants (S6A, S7, S10 and S11 Figs), but combining H3 ubiquitin-deficient mutants with *asf1*Δ or *rtt109*Δ mutants was lethal or near lethal, precluding their assessment upon exposure to UV (S6B, S7 and S11 Figs). In contrast, loss of *HIF1* did not lead to further sensitivity to UV when combined with the H3 mutants (S6A, S7, S10 and S11A Figs). Loss of *HAT1* in combination with single H3 mutants also did not dramatically affect growth in response to UV relative to single histone mutants except for partial suppression of growth defects when combined with H3 K122A mutants (S6C and S7 Figs). Synthetic sensitivity to UV was not observed for histone mutants combined with loss of *SAS2* or *DOT1* (S6B, S6C, S10 and S11 Figs), despite Dot1p and H3 K79 being important for efficient Global Genomic Repair of UV-mediated lesions [92–94].

Previously, Asf1p (via promoting H3 K56ac [95]) and CAF-1, but not Rtt106p, have been implicated in chromatin assembly during recovery from DSB repair [74,96]. *RTT101* has been reported to be required for checkpoint recovery via the same pathway as *ASF1*, but not for assembling chromatin *per se* after DSB repair [74]. As Rtt101p-mediated H3 K122ub promotes association of H3/H4 with Rtt106p, we also assessed sensitivity to DNA alkylation (MMS [97]), double stranded DNA breaks (Zeocin [98]), and replication stress via inhibition of ribonucleotide reductase (hydroxyurea, HU [99]) of H3 K122R and H3 K122Q mutants in *cac1*Δ mutants expressing vector alone, *CAC1*, or the phospho-mutants *cac1 S238*,*503D* or *cac1 S238*,*503A* (S8B Fig) and *cac1 S501*,*503D* or *cac1 S501*,*503A* (S9B Fig). S238 and S503 lie within Cdc7p-consensus phosphorylation sites on Cac1p [50,100–103], and S501 and 503 of Cac1p become phosphorylated in response to DNA damage, but the significance of these phosphorylation events is unknown [104]. Our analyses revealed that H3 K122R and H3 K122Q mutants were hypersensitive to MMS, Zeocin, and hydroxyurea (S8B and S9B Figs) in addition to UV (S8A and S9A Figs) relative to wild-type H3. This sensitivity increased in the absence of *CAC1*, but was suppressed by exogenous expression of *CAC1*, as well as the *cac1* phospho-mutants. Similarly, H3 K121,122,125R mutants were hypersensitive to MMS and HU relative to wild-type, and this sensitivity to MMS increased in the absence of *CAC1* (S12 Fig). Synthetic interactions during replication stress could not be ascertained under conditions tested due to the hypersensitivity of H3 K121,122,125R mutants to HU. Together, these results are consistent with these repair-related defects of the H3 ubiquitin mutants being associated with a *CAC1*-independent pathway. However, as *cac2*Δ and *cac2*Δ *rtt106*Δ mutants are similarly sensitive to DSBs [74], this H3 K122R/Q-related hypersensitivity to zeocin is not likely due to defects in Rtt106p-mediated chromatin assembly during checkpoint recovery from DSBs.

### Defects in coordinating activities on leading and lagging strands during DNA replication restore silencing at HMRae**

To understand why Rtt101p was important for preventing silent chromatin from forming at *HMR****a***e** (Fig 4G), we next turned our attention to identifying Rtt101p interacting factors that also functioned in this pathway. The cullin Rtt101p requires the adaptor Mms1p to bind H3 *in vitro* and *in vivo*, but neither binding nor ubiquitination of H3 by Rtt101p/Mms1p *in vitro* requires the Rtt101p/Mms1p-associated substrate receptor Mms22p [34]. As *RTT101* and H3 ubquitination fell in different pathways with respect to silencing at *HMR****a***e**, we next assessed the impact of loss of *MMS1* or *MMS22* on silencing at *HMR****a***e**. Like *rtt101*Δ mutants, but in contrast to *rtt106*Δ and H3 K122A or R mutants, silencing was restored at *HMR****a***e** in cells lacking either *MMS1* or *MMS22* (Fig 5A) in a *SIR2*-dependent manner (Fig 5B). These observations supported the possibility that an H3 ubiquitination-independent function of Rtt101p influenced silencing at *HMR****a***e**. Rtt101p/Mms1p also forms a protein complex with Orc5p, however, *orc5-1* mutants do not restore silencing at *HMR****a***e** [105]. In addition, Rtt101p/Mms1p/Mms22p also interacts with several factors associated with the replication fork, including the ortholog of human AND-1, Ctf4p [70–72]. As Ctf4p promotes the coordination of leading and lagging strands during replication [106–109], and the transfer of parental histones to lagging strands during replication [109], we tested the impact of loss of *CTF4* on silencing at *HMR****a***e** via patch mating assays. Like in *mms22*Δ mutants, silencing at *HMR****a***e** was restored in cells lacking *CTF4* as well as *CTF4* plus *CAC1* (Fig 5C), although *ctf4*Δ *cac1*Δ mutants also exhibited a severe negative interaction for growth (Fig 5D) (see also [110–112]). Thus, in the *rtt101*Δ mutants, perturbations to events at the replication fork other than H3 K122ub-dependent Rtt106p-mediated chromatin assembly likely contributed to defects in H4 K16ac and the restoration of silencing at *HMR****a***e**. Determining whether this could reflect a leading or lagging strand-specific defect awaits future studies.

### *RTT101* is required for Asf1p-CAF-1 interaction and efficient H4 K16ac at HMRae**

To assess further the impact of loss of *RTT101* on CAF-1 function, we tested whether *RTT101* was required for interactions between Asf1p and Cac1p *in vivo*. In this analysis, yeast expressing Asf1-GFPp, Asf1-GFPp and Cac1-mCherryp versus *rtt101*Δ mutants expressing Asf1-GFPp and Cac1-mCherryp were analyzed by FLIM-FRET. The reduction of lifetime of Asf1-GFPp in cells expressing both Asf1-GFPp and Cac1-mCherryp was dependent on *RTT101* in small budded cells, as was the FRET interaction between Asf1-GFPp and Cac1-mCherryp (Fig 6).

**Fig 7:**
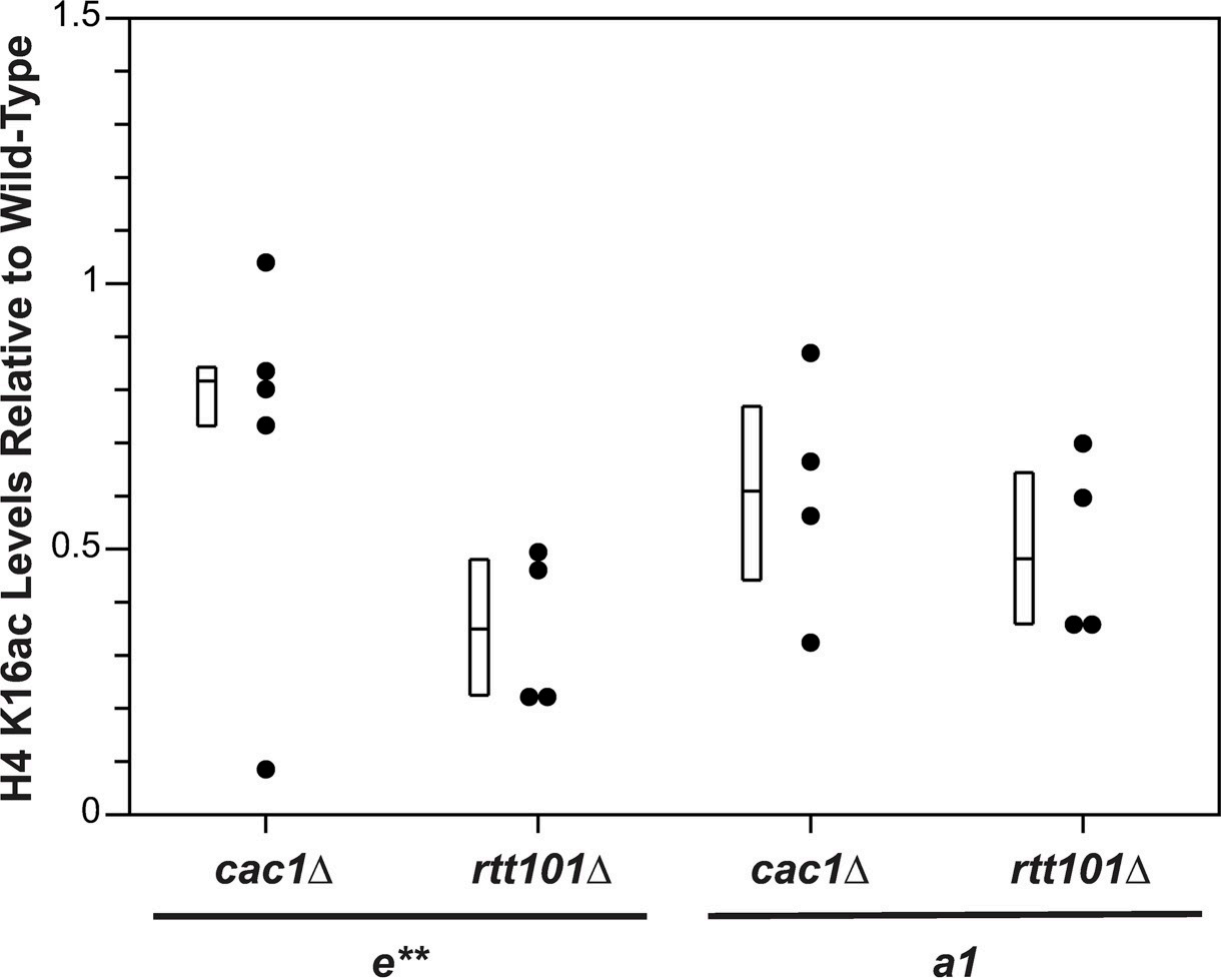
H4 K16 is hypoacetylated at HMRae** in cells lacking *RTT101*. H4 K16ac levels at the e** silencer and ***a1*** in *MAT*α *HMR****a***e** *sir2*Δ strains with the indicated genotypes were monitored by ChIP. The efficiency of coprecipitation of DNA with H4 K16ac antibodies was determined relative to that with anti-H3 antibodies, and normalized to yeast lacking *SIR2*, which was set to 1. Data were calculated as 2^[(H4 K16ac C_T_ - H3 C_T_)_WT_ - (H4 K16ac C_T_ - H3 C_T_)_genotype_]^. Mean ± STD, n = 4 or 5. Each mutant was hypoacetylated relative to WT at both e** and ***a1*** (p = 0.08 for *cac1*Δ at e**; p = 0.007 for all other samples and loci; Wilcoxon Rank Sum Test).

**Fig 8:**
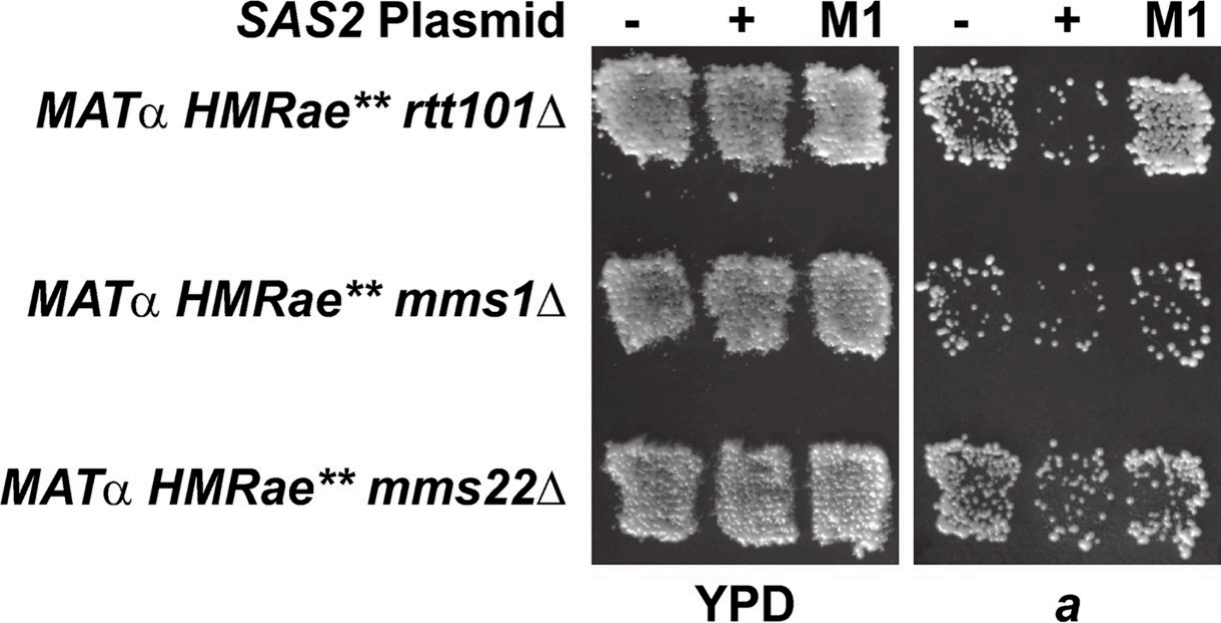
Overexpression of catalytically active Sas2p disrupts silencing at HMRae** in *rtt101*Δ, *mms1*Δ, or *mms22*Δ mutants. Patch Mating Assays. Cells with the indicated genotypes were analyzed as in Fig 1.

One prediction from this observation was that this defect in interaction between Asf1p-Cac1p in the absence of *RTT101* would lead to defects in CAF-1 pathway-dependent acetylation of H4 K16. Therefore, we monitored H4 K16ac levels at *HMR****a***e** by chromatin immunoprecipitation (ChIP) in *cac1*Δ *sir2*Δ or *rtt101*Δ *sir2*Δ strains relative to *sir2*Δ strains. (Use of *sir2* mutants enabled evaluation of Sir2p-independent effects on histone acetylation at *HMR****a***e**.) Similar to *cac1*Δ mutants, e** and ***a1*** were hypoacetylated in *rtt101*Δ mutants relative to wild-type (Fig 7; p = 0.007). However, like *cac1*Δ, and *sas2*Δ mutants, the cell cycle distribution of logarithmic cultures of *rtt101*Δ mutants was similar to wild-type (S13 Fig). Thus, hypoacetylation of H4 K16 at *HMR****a***e** in the *rtt101*Δ mutants was not simply due to an enrichment of cells outside of S phase. Instead, these results were consistent with restoration of silencing in the *rtt101*Δ mutants having been facilitated, at least in part, by hypoacetylation of histone H4 K16 at *HMR****a***e** via perturbations to a CAF-1-dependent pathway. Consistent with this model, silencing at *HMR****a***e** in *rtt101*Δ, *mms1*Δ and *mms22*Δ mutants was disrupted upon overexpression of *SAS2*, but not catalytically inactive sas2-M1p (Fig 8) [51,68].

## Discussion

Here we have illustrated processing of histones through different pathways within the replication-coupled chromatin assembly network enables the deposition of histones with distinct modification patterns in chromatin, by highlighting a CAF-1-dependent pathway that prevents formation of new epigenetic states at an ectopic site via deposition of H4 K16ac. We demonstrated that although both Cac1p and Rtt106p interacted with Asf1p in a *RTT109*-dependent manner (Figs 2 and 3), CAF-1 and Rtt106p functioned in distinct pathways as highlighted by their different effects on silencing and H4 K16ac (Figs 1, S5 and S1 Figs and Table 1), plus synthetic interactions with histone H3 ubiquitination mutants (S5–S12 Figs). Loss of *CAC1* restored silencing at *HMR****a***e** and led to a decrease in H4 K16ac in chromatin, but H4 K16ac remained at wild-type levels in *RTT106* mutants (Figs 1C and 5C, Table 1, and S1 Fig, see also [49,50]). Consistent with restoration of silencing at *HMR****a***e** in the *cac1*Δ mutants being due to hypoacetylation of H4 K16 (Table 1 and S1 Fig, [49]), this phenotype could be suppressed upon overexpression of *SAS2* [50]. Similarly, loss of *ASF1* or *RTT109* restored silencing at *HMR****a***e** (Figs 1C and 4A). This phenotype was suppressed by overexpression of *SAS2* (Fig 4B and 4C, [50]), and *asf1*Δ and *rtt109*Δ mutants exhibited defects in H4 K16ac in chromatin (Table 1 and S1 Fig, [49]). Together, these data imply that acetylation of H4 K16 during chromatin assembly occurs on histone H4 processed through a *CAF-1-*mediated pathway, but not a *RTT106*-dependent pathway (see also [48,51,52].

Our results also indicated for the first time the cullin Rtt101p affects not only the *RTT106*- dependent pathway, but also a *CAC1*-dependent pathway within the replication-coupled chromatin assembly network in yeast, and does so via different mechanisms. We observed that H3 ubiquitin mutants, like *rtt106*Δ mutants, failed to restore silencing at *HMR****a***e** (Fig 4F–4H, and Table 3). In synthetic interaction analyses of growth, silencing, and UV sensitivity, H3 K122A/R mutants primarily exhibited negative synthetic interactions with *cac1*Δ mutants but not with *rtt106*Δ mutants (S5 and S6 Figs). In contrast, loss of *RTT101* or its binding partners Mms1p-Mms22p restored *SIR*-dependent silencing at *HMR****a***e** (Figs 4G and 5B), likely through disrupting an interaction between Asf1p and Cac1p (Fig 6), which led to hypoacetylation of H4 K16 at *HMR****a***e** (Fig 7). Further, we provide evidence that this latter pathway likely also involved the Mms22p interacting factor Ctf4p (Fig 5C), which coordinates leading and lagging strand synthesis at the DNA replication fork [106–109]. Our synthetic interaction analyses indicated H3 mutants with defects in ubiquitination exhibited differing genetic interactions with mutants in the replication-coupled chromatin assembly network for silencing, growth and DNA damage sensitivity (S5–S11 Figs). Thus, not only ubquitination, but also the charge state of H3 K122, or another attribute of this region of H3 altered by K122 mutations, played important roles in these processes.

### Model for the replication-coupled chromatin assembly network

**Fig 9:**
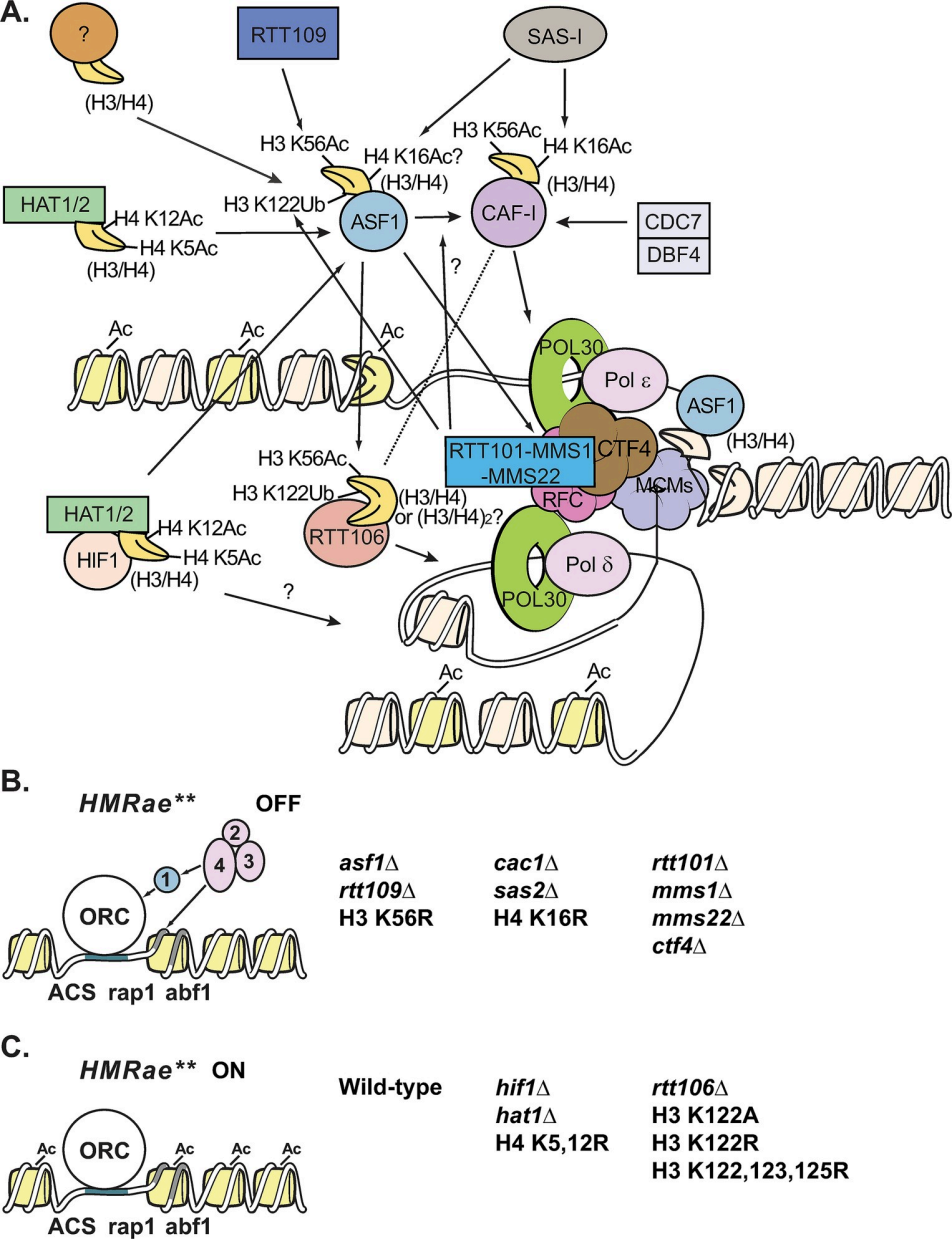
Model of the Replication-Coupled Chromatin Assembly Network and silencing. **A)** Replication-Coupled Chromatin Assembly. Newly synthesized H3/H4 dimers (yellow) are acetylated at H4 K5 and 12 by the HAT-B complex (Hat1p and Hat2p (dull green)) or the NuB4 complex (HAT-B plus Hif1p (peach)) and are transported to the nucleus and can be transferred to Asf1p (light blue). Asf1p can also acquire new H3/H4 dimers by a HAT-B or NuB4-independent method(s) (tan). Rtt109p (dark blue) binds to H3/H4 dimers bound by Asf1p, and acetylates H3 K56, which promotes binding of H3/H4 to the CAF-1 complex (magenta) or to Rtt106p (coral). Sas2p of the SAS-I complex (grey) binds CAF-1 (and/or Asf1p) and acetylates H4 K16. H4 K16ac is also promoted by Cdc7p/Dbf4p (light grey), likely through phosphorylation of Cac1p at S238 and S503. Rtt106p preferentially binds newly synthesized H3/H4 containing H3 K56ac or Rtt101p-dependent H3 K122ub. Rtt106p can also interact with the Cac1p subunit of CAF-1. PCNA (Pol30p, bright green) tethers DNA polymerases (Polδ and Polε, light pink) to the replication fork, and is loaded onto DNA by RFC (dark pink). CAF-1 and Asf1p associate with the replication fork through interactions between Cac1p and PCNA, Asf1p and RFC (dark pink), and possibly via an interaction between CAF-1 and Asf1p that is promoted by Rtt101p (medium blue). Rtt101p-Mms1p-Mms22p (medium blue) may be localized to the replication fork via interactions between Mms22p and Ctf4p (brown). Assembly of new (yellow) and/or parental (beige) histones behind the fork is facilitated by FACT (not shown) as well as the MCM helicase (purple), CAF-1, Rtt106p, Asf1p, likely Hif1p, and additional factors. **B)** Defects in an assembly pathway that mediates H4 K16ac during DNA replication results in silencing at *HMR****a***e**. Cells lacking *RTT109*, *ASF1*, *CAC1*, SAS-I subunits, *RTT101*, *MMS1*, *MMS22* or *CTF4*, or expressing H3 K56R or H4 K16R all restore silencing at *HMR****a***e** (“OFF”). **C)** Defects in other pathways for replication-coupled assembly do not promote silencing at *HMR****a***e**. Loss of *HIF1*, *HAT1*, or expression of H4 K5,12R, or loss of *RTT106*, or expression of H3 K122A, H3 K122R or H3 K121,122,125R does not restore silencing at *HMR****a***e** (“ON”). 1, 2, 3, and 4 are Sir1p, Sir2p, Sir3p, and Sir4p, respectively. Please see text for additional details.

Together, our results support a model (Fig 9) in which distinct modification patterns on histones are created during replication-coupled chromatin assembly based on which pathway within the replication-coupled chromatin assembly network has been used to process histones. This enables the assembly of nucleosomes containing distinct, pathway-dependent, modification patterns. Some of these modifications, such as Hat1p-dependent H4 K5ac and H4 K12ac, along with histone binding by Hat1p, promote nuclear import and chromatin assembly [44,45,113,114], and likely occur very early during processing of newly synthesized histones. Consistent with this, H4 K5ac and H4 K12ac is present in both soluble and chromatin fractions [115] as well as on H3/H4 bound by downstream factors such as Asf1p [34], ASF1B in mammals [116], and CAF-1 [82]. Others, such as Rtt109p-dependent H3 K56ac and Rtt101p-dependent H3 K122ub function, at least in part, to direct histones H3/H4 down specific processing pathways via promoting or disrupting interactions between histones H3/H4 and certain chaperones such as CAF-1 or Rtt106p (Figs 2 and 3) [4,34]. *RTT101* or H3 K122ub were not required for H3 K56ac (Fig 4D) or H4 K5,12ac [34,69]. In contrast, and consistent with H3 K56ac being upstream of H3 K122ub, co-precipitation of Rtt101p with H3 is reduced in cells lacking *RTT109* or *ASF1* [34], Rtt101p-Mms1p complexes preferentially bind H3 acetylated at K56 *in vitro*, and mutants that lack H3 K56ac have reduced levels of ubiquitinated H3 [34]. H3 K122ub appears to promote preferential binding of H3/H4 to Rtt106p, but not to CAF-1 [34], and therefore may serve to select this modified subpopulation of H3/H4 for processing and deposition along a Rtt106p-dependent assembly pathway.

In this model (Fig 9), Sas2p-dependent acetylation of H4 K16 occurs during a step downstream of Rtt109p-dependent acetylation of H3 K56 while H3/H4 are being processed through a CAF-1-mediated pathway within the network, and cells lacking factors in this pathway have defects in H4 K16ac (Table 1, Fig 7 and S1 Fig, see also [49,50]). Therefore, H4 hypoacetylated at K16 would become loaded onto the chromosome at *HMR****a***e** during replication when this pathway is defective, thereby creating high affinity binding sites for Sir proteins, enabling their recruitment, and increasing the probability of the formation of silent chromatin (Figs 1, 4, 5 and 9B, see also [49,50]). Here, we provide evidence that this pathway also involves Rtt101p (Figs 4G, 5B and 7), which promotes interaction between CAF-1 and Asf1p (Fig 6), as well as Rtt101p’s binding partners Mms1p and Mms22p, plus Mms22p’s binding partner Ctf4p (Fig 5A–5C), but not Rtt101p-dependent H3 K122ub (Figs 4F, 4H, 9A, 9C and Table 3). In addition, we previously demonstrated this pathway involves the cell cycle-dependent kinase Cdc7p, likely via regulating a Cac1p function related to Sas2p-mediated H4 K16ac [50]. We speculate Ctf4p plays a role in coordinating the assembly of nucleosomes containing H4 K16ac at the replication fork [109], through a process involving its association with Rtt101p/Mms1p/Mms22p (see also [70,71]), but testing this model directly awaits further studies. If correct, this would imply that transfer of histones H3/H4 from Asf1p to CAF-1 or Rtt106p typically occurs at the replication fork itself, which is consistent with our observations that Asf1p and PCNA interact in live cells (S3 Fig). This overall model is also supported by our previous observations that Rtt109p and SAS-I interact with wild type PCNA in live cells, but not with pol30p mutants with defects in *ASF1-* or CAF-1-dependent pathways [49] as well as the others’ observations that Asf1p interacts with the PCNA loader RFC [55], CAF-1 interacts with PCNA [35,67,117–119], Asf1p binds Cac2p weakly *in vitro* [67], and Rtt106p interacts with the PCNA unloader Elg1p [120].

In contrast to the Rtt109p-H3 K56-Asf1p-CAF-1-mediated assembly pathway discussed above, in this model, H4 K16ac by Sas2p does not require a pathway that involves directing H3/H4 from Asf1p to Rtt106p via Rtt109p (Figs 1C and 2 and Table 1) [4] and Rtt101p-dependent H3 K122ub (Fig 4 and Table 3) [34] during replication-coupled chromatin assembly, despite Rtt106p being able to associate with CAF-1 through Cac1p [1,35] (Fig 9A–9C). Sas5p continued to associate with chromatin upon deletion of *RTT106* as well as *CAC1* or *ASF1* (Table 2 and S2 Fig). As SAS-I co-precipitates with Cac1p as well as Asf1p [51,52], SAS-I may have been recruited efficiently to chromatin in the presence of only one binding partner, albeit in a manner in which either H4 was no longer efficiently acetylated at K16 or incorporated into chromatin. Consistent with this notion, association of Sas2p with the NTS of the rDNA locus is only lost in *asf1*Δ *cac1*Δ double mutants [52].

### H3 K122, *RTT101*, and chromatin assembly

At least seven modifications to H3 K122, in addition to ubiquitination, have been reported for different organisms [75–80]. Whether H3 K122 can similarly be modified in budding yeast [121], and what the biological functions of these modifications are have largely not been established. The H3 K122 residue lies near the interaction surface between Asf1p and H3-H4 [19], the tetramerization surface of H3-H4 [19,122], as well as between histone-DNA contacts [123]. Therefore, the charge or modification state of this residue might affect several interactions, including those that occur outside of replication-coupled chromatin assembly. For example, H3 K122Q results in the loss of a salt bridge between K122 and DNA, and may weaken the interaction between histone octamers and DNA [124], which is supported by the silencing defects in H3 K122Q mutants (S5 Fig, see also [84]). In mammals, H3 K122ac weakens interactions between the histone octamer and DNA *in vitro* [123] and is associated with increased transcription *in vitro* and *in vivo* [79]. However, H3 K122ac does not affect reconstitution of nucleosomes from purified histones onto DNA containing a nucleosome positioning sequence [124], indicating that H3 K122ac does not inhibit nucleosome formation *per se*. Nucleosomes containing H3 K122ac also behave similarly to unmodified nucleosomes in gel shift analyses and sucrose gradient centrifugation, indicating H3 K122ac does not greatly affect nucleosome stability *in vitro* [123].

We tested the genetic interactions between H3 K122A/R/ and Q mutants with different chromatin assembly factor mutants to gain a better understanding of the impact of *RTT101*-dependent ubiquitination of H3 and the charge state at H3 K122 on chromatin assembly pathways. Interestingly, in our synthetic interaction analyses of H3 ubiquitin mutants and factors involved in the replication-coupled chromatin assembly network, we observed variable negative synthetic interactions for silencing (S5 Fig) as well as growth and DNA damage sensitivity (S5–S12 Figs) that further supported a model in which Cac1p and Rtt106p function in separate pathways and that the charge status of H3 K122, not necessarily ubiquitination alone, affected histone deposition. Consistent with this, we also observed severe negative synthetic interactions for growth as well as DNA damage sensitivity between H3 ubiquitin mutants and loss of *ASF1* or *RTT109* (S6, S7 and S11 Figs). These double mutants are expected to have defects in both *RTT106*- and CAF-1-dependent pathways. However, as *rtt106*Δ *caf1*Δ mutants do not similarly exhibit severe growth defects [23], this region of H3 likely has an additional function(s) that become critical in cells lacking *ASF1* or *RTT109*, and may reflect the existence of an additional assembly pathway within the network that functions in parallel to Asf1p/Rtt109p and requires H3 to be appropriately modified in this region. Consistent with our observations, previous studies have also demonstrated that H3 K122A/R/ and Q mutants exhibit varied phenotypes [19,34,84,125].

In human cells, loss of the E3 ubiquitin ligase Cul4 leads to decreased association of H3 with p150 of CAF-1 as well as the Rtt106p-like protein Daxx [34]. Consistent with conservation of a functional relationship between the cullin and both assembly factors, we provide evidence Rtt101p impacts chromatin assembly through a CAF-1-dependent pathway in addition to a Rtt106p-dependent pathway in budding yeast. Loss of *RTT101* resulted in a defect in Asf1p-Cac1p interaction (Fig 6), and in H4 K16ac at *HMR****a***e** (Fig 7) as well as the restoration of silencing at *HMR****a***e** (Figs 4G and 5B). Surprisingly, however, co-precipitation of H3 with Cac2p does not require *RTT101* [34]. The reason for this apparent discrepancy is currently unclear. It is possible that, in our studies, loss of *RTT101* had further reduced the stability of a transient Asf1p-H3/H4-CAF-1 ternary complex, which prevented detection of the interaction by FLIM-FRET as well as acetylation of H4 K16 by SAS-I. Or, loss of *RTT101* may have altered the confirmation of an Asf1p-H3/H4-CAF-1 complex, which disrupted acetylation of H4 K16 by SAS-I as well as altered the conformation of Asf1-GFPp and Cac1-mCherryp such that their fluorophores were now greater than 10 nm apart and unable to support a FRET interaction. Either scenario could have resulted in hypoacetylation of H4 K16 without eliminating the transfer of histones H3/H4 from Asf1p to CAF-1. Alternatively, in the absence of *RTT101*, CAF-1 could have acquired histones H3/H4 in an *ASF1*-independent manner. Clarifying how Rtt101p contributes to the CAF-1-dependent chromatin assembly pathway awaits future studies, but *RTT101* clearly has functions that impact chromatin assembly during DNA replication in addition to the ubiquitination of H3 K122.

### Functions of HAT-B and NuB4 in the replication-coupled chromatin assembly network

As the HAT-B complex and the NuB4 complex interact with Asf1p [41,42,47,126], and histone H4 copurifying with Asf1p or CAF-1 contains the HAT-B-mediated modifications H4 K5,12ac [44–46,116], HAT-B (and NuB4) likely have a function(s) upstream of a Rtt109p-H3 K56-Asf1p-CAF-1-mediated assembly pathway. However, *hat1*Δ and *hif1*Δ mutants are viable, *HIF1* (and, therefore, NuB4) is not required for H3K56ac (Fig 4E), and the negative synthetic interactions between, e.g., H3 K122Q and *hat1*Δ or *hif1*Δ mutants are generally less severe than between H3 K122Q and *asf1*Δ or *rtt109*Δ mutants (S6 Fig). Thus, newly synthesized H3/H4 must also be able to enter the Asf1p/Rtt109p/CAF-1/Sas2p-mediated assembly pathway through a HAT-B or NuB4-independent mechanism. Consistent with this notion, neither *hat1*Δ or *hif1*Δ mutants nor H3 K5,12R mutants could restore silencing at *HMR****a***e** (Fig 1 and [52]), in contrast to *rtt109*Δ, *asf1*Δ, *cac1*Δ, *sas2*Δ, H3K56R or H4 K16R mutants (Figs 1, 9B and 9C). Moreover, H4 K5, 12R mutants synthetically interact with *rtt109*Δ and H3 K56R mutants under normal growth conditions and in the presence of DNA damage agents [4]. In addition, our results are consistent with the *RTT106*-dependent pathway not requiring *HIF1*, and Hif1p functioning in at least one additional chromatin assembly pathway that is CAF-1- or *RTT106*-independent. In contrast to *cac1*Δ mutants, *hif1*Δ and *hat1*Δ mutants, like *rtt106*Δ mutants, do not exhibit negative synthetic interactions with H3 K122A mutants for silencing *HMR*::*ADE2* (S5D and S5E Fig), and yet synthetic interactions of *hif1*Δ versus *cac1*Δ or *rtt106*Δ mutants plus H3 K122,125R or H3 K121,122,125R mutants do not phenocopy each other (S10–S12 Figs). We also observed that negative synthetic interactions of H3 ubiquitin mutants with *hat1*Δ mutants were generally more severe than those with *hif1*Δ mutants (S6, S10, S11 and S12 Figs), implying that the Hat1p-Hat2p-containg HAT-B complex has one or more functions independent of the Hat1p-Hat2p-Hif1p-containing NuB4 complex [see also [114] and references within]. Future studies will be required to clarify the relationships between Hif1p or Hat1p and other factors within this replication-coupled chromatin assembly network.

### Summary

Collectively, our findings reported here demonstrate the potential for different histone modifications to regulate usage of different replication-coupled chromatin assembly pathways and underlie the importance of nucleosome assembly pathways in customizing the histone code created along the genome during replication. Why multiple different histone deposition pathways exist during replication is unclear, but our data supports a model in which these different pathways are responsible for the deposition of distinct modified forms of histones that, in turn, can influence the establishment, maintenance or inheritance of active or silenced epigenetic states, in a locus-, and potentially, sister chromatid-specific manner. Further, the ability of budding yeast to survive in the absence of individual or multiple histone chaperones, and replication-couple histone modifications, implies that, despite their having evolved distinct specialized functions, the histone deposition pathways remain partially functionally redundant for those aspects of chromatin assembly critical for viability [15,127,128].

## Supporting information

S1 Table Yeast strains used in this study. (DOCX)

S2 Table Plasmids used in this study. (DOCX)

S3 Table Oligos used in this study. (DOCX)

S4 Table Numerical Data. (XLSX)

S1 Fig *rtt109*Δ mutants have defects in chromatin-associated H4 K16ac. Immunoblot analysis of H4 K16ac and H3 levels in chromatin fractions isolated from indicated genotypes. Immunoblot shown is representative of three biological replicates used to generate quantification data in Table 1.
(TIF)

S2 Fig Sas5p associates with chromatin in chromatin assembly network mutants. Immunoblot analysis of Sas5-YFP and H3 levels (loading control) in chromatin fractions isolated from indicated genotypes with Ponceau S staining of total protein levels. Immunoblot shown is representative of four biological replicates used to generate quantification data in Table 2.
(TIF)

S3 Fig Asf1p interacts with PCNA *in vivo*. **A)** Confocal fluorescence lifetime images of GFP in small-budded live cells expressing the fluorescently-tagged proteins as indicated. White scale bars are equivalent to 5μm. FLIM scale bar: 1 nanosecond, blue; 3 nanoseconds, red. **B)** The average lifetime of GFP in indicated strains. Error bars represent the standard deviation of ten FLIM measurements taken for each genotype. **C)** FRET efficiency of indicated strains.
(TIF)

S4 Fig Overexpression of *SAS2* in *rtt109*Δ mutants results in variegated suppression of *SIR2*-dependent silencing at HMRae**. Individual colonies of clones with the indicated genotypes were expanded and grown as individual patches on minimal medium supplemented for auxotrophic markers (YM-LEU) plates at 30°C overnight, then were replica plated onto either a *MAT****a*** lawn (JRY2726) on minimal medium, or rich medium (YPD), and were grown at 30° for two days. Cells grown on the YM-LEU plate were also replica plated onto a YPD plate containing 300 μM dihydrocoumarin (DHC) to inhibit Sir2p [129–131], grown at 30°C overnight, and then replica plated as above.
(PDF)

S5 Fig Genetic Interactions between H3 K122 mutants and replication-coupled chromatin assembly pathway factors contribute to defects in silencing *HMR*::*ADE2*. **A)** Map of *HMR*::*ADE2* Reporter. **B-E)** Genetic interactions between H3 K122R **(B)**, H3 K122Q **(C)** or H3 K122A **(D** and **E)** and chromatin assembly pathway mutants. Cells with the indicated genotypes were grown in YPD at 30°C overnight, then spotted onto CSM plates in ten-fold serial dilutions, and grown for two days at 30°C. Cells were then incubated at 4°C for four days prior to imaging.
(TIF)

S6 Fig Genetic interactions between H3 K122R and Q mutants and replication-coupled chromatin assembly pathways factors contribute to temperature-sensitive growth defects plus sensitivity to UV. **A-C)** Genetic interactions between H3 K122R or H3 K122Q and chromatin assembly pathway mutants. Cells with the indicated genotypes were grown in YPD at 30°C overnight, then spotted onto CSM or YPD plates in ten-fold serial dilutions, and grown at the temperature indicated for two days, or were then treated with either 50 or 100 J/m^2^ of UV light and grown at 30°C for two days. Color images of 30°C plates of some H3 K122Q mutants are shown in S5 Fig.
(TIF)

S7 Fig Genetic interactions between H3 K122A mutants and replication-coupled chromatin assembly pathways factors contribute to temperature-sensitive growth defects plus sensitivity to UV. Strains were analyzed as outlined in S6 Fig legend. Color images of 30°C plates are shown in S5 Fig.
(TIF)

S8 Fig Genetic Interactions between H3 K122R or H3 K122Q and *cac1* mutants are suppressed by expression of exogenous *CAC1*, *cac1 S238*,*503D* and *cac1 S238*,*503A*. **A)** Genetic interactions sensitive to temperature or exposure to UV. **B)** Genetic interactions upon exposure to DNA damaging agents. Yeast with the indicated genotypes were grown in YPD at 30°C overnight, then spotted onto complete supplement medium (CSM) or YPD plates in ten-fold serial dilutions, and grown at the temperature indicated for two days, or were then treated with 50 or 100 J/m^2^ of UV **(A)**, or the indicated amounts of methyl methanesulfonate (MMS), Zeocin, or hydroxyurea (HU) **(B)**, and grown at 30°C for two days.
(TIF)

S9 Fig Genetic Interactions between H3 K122R or H3 K122Q and *cac1* mutants are suppressed by expression of exogenous *CAC1*, *cac1 S501*,*503D* and *cac1 S501*,*503A*. **A)** Genetic interactions sensitive to temperature or exposure to UV. **B)** Genetic interactions upon exposure to DNA damaging agents. Yeast were assayed as outlined in S5 Fig.
(TIF)

S10 Fig Genetic Interactions between H3 K122,125R mutants and factors involved in replication-coupled chromatin assembly contribute to temperature- and cold-sensitive growth defects plus sensitivity to UV. Cells with the indicated genotypes were grown in YPD at 30°C overnight, then spotted onto YPD plates in ten-fold serial dilutions, and grown at the temperature indicated for two days, or were then treated with either 75 or 100 J/m^2^ of UV light and grown at 30°C for two days.
(TIF)

S11 Fig Genetic Interactions between H3 K121,122,125R mutants and factors involved in replication-coupled chromatin assembly contribute to temperature- and cold-sensitive growth defects plus sensitivity to UV. *cac1*Δ, *rtt106*Δ, *hif1*Δ, *hat1*Δ, *asf1*Δ, *sas1*Δ, *dot1*Δ **(A)** or *rtt109*Δ mutants **(B)** relative to wild-type were assayed as outlined in S7 Fig.
(TIF)

S12 Fig Genetic Interactions between H3 K121,122,125R mutants and factors involved in replication-coupled chromatin assembly upon exposure to MMS or HU. Cells with the indicated genotypes were grown in YPD at 30°C overnight, then spotted onto YPD plates in ten-fold serial dilutions, in the absence or presence of the indicated amounts of MMS or HU at 30°C for two days.
(TIF)

S13 Fig *rtt101* mutants have a normal cell cycle distribution. Flow Cytometry. Yeast with the indicated genotypes were grown logarithmically in YPD at 30°C prior to harvesting to assess their cell cycle distribution by Flow Cytometry.
(TIF)
