# Supplementary material for: CAF-1 and Rtt101p function within the replication-coupled chromatin assembly network to promote H4 K16ac, preventing ectopic silencing
Source: PLoS Genet. 2020 Dec 7;16(12):e1009226. doi: 10.1371/journal.pgen.1009226 (PMC7746308; doi:10.1371/journal.pgen.1009226)
Supplement: S1 Table — (DOCX) [file pgen.1009226.s001.docx]

| **S1 TABLE. Yeast strains used in this study.** | |  | |
| --- | --- | --- | --- |
| **Strain** | **Genotype** | | **Source** |
| JRY2726 | *MAT****a*** *his4* | | P. Schatz |
| BY4741 | *S288C MAT****a*** *his3*Δ*1 leu2*Δ*0 met15*Δ*0 ura3*Δ*0* | | [1] |
| W303 | *MATα ade2-1 his3-11,15 leu2-3,112 trp1-1 ura3-1 can1-100* | | R. Rothstein |
| AKY1677 | *MATα HMR****a****e** ade2-1 his3-11,15 leu2-3,112 trp1-1 ura3-1 can1-100* | | [2]^1^ |
| AKY2399 | *MATα HMR****a****e** ade2-1 his3-11,15 leu2-3,112 trp1-1 ura3-1 can1-100 cac1*Δ*::KanMX* | | [2]^1^ |
| AEY2363 | *MATα HMR****a****e** ade2-1 his3-11,15 leu2-3,112 trp1-1 ura3-1 can1-100 asf1*Δ*::KanMX* | | [3]^1^ |
| AEY474 | *MATα HMR****a****e** ade2-1 his3-11,15 leu2-3,112 trp1-1 ura3-1 can1-100 sas2*Δ*::KanMX* | | [3]^1^ |
| AKY2726 | *MATα HMR****a****e** ade2-1 his3-11,15 leu2-3,112 trp1-1 ura3-1 can1-100 hif1*Δ*::KanMX* | | This Study |
| AKY2730 | *MATα HMR****a****e** ade2-1 his3-11,15 leu2-3,112 trp1-1 ura3-1 can1-100 rtt106*Δ*::KanMX* | | This Study |
| AKY2781 | *MATα HMR****a****e** ade2-1 his3-11,15 leu2-3,112 trp1-1 ura3-1 can1-100 hat1*Δ*::KanMX* | | This Study |
| AKY6416 | *MATα HMR****a****e** ade2-1 his3-11,15 leu2-3,112 trp1-1 ura3-1 can1-100 rtt109*Δ*::KanMX* | | This Study |
| AKY9033 | *MATα HMR****a****e** ade2-1 his3-11,15 leu2-3,112 trp1-1 ura3-1 can1-100 rtt109*Δ*::KanMX sir2*Δ*::TRP1* | | This Study |
| AKY6607 | *MATα HMR****a****e** ade2-1 his3-11,15 leu2-3,112 trp1-1 ura3-1 can1-100 rtt101*Δ*::KanMX* | | This Study |
| AKY9053 | *MATα HMR****a****e** ade2-1 his3-11,15 leu2-3,112 trp1-1 ura3-1 can1-100 rtt101*Δ*::KanMX sir2*Δ*::LEU2* | | This Study |
| AKY6619 | *MATα HMR****a****e** ade2-1 his3-11,15 leu2-3,112 trp1-1 ura3-1 can1-100 mms1*Δ*::KanMX* | | This Study |
| AKY9073 | *MATα HMR****a****e** ade2-1 his3-11,15 leu2-3,112 trp1-1 ura3-1 can1-100 mms1*Δ*::KanMX sir2*Δ*::TRP1* | | This Study |
| AKY6621 | *MATα HMR****a****e** ade2-1 his3-11,15 leu2-3,112 trp1-1 ura3-1 can1-100 mms22*Δ*::KanMX* | | This Study |
| AKY9077 | *MATα HMR****a****e** ade2-1 his3-11,15 leu2-3,112 trp1-1 ura3-1 can1-100 mms22*Δ*::KanMX sir2*Δ*::TRP1* | | This Study |
| AKY9058 | *MATα HMR****a****e** ade2-1 his3-11,15 leu2-3,112 trp1-1 ura3-1 can1-100 ctf4*Δ*::KanMX* | | This Study |
| AKY9082 | *MATα HMR****a****e** ade2-1 his3-11,15 leu2-3,112 trp1-1 ura3-1 can1-100 cac1*Δ*::KanMX ctf4*Δ*::KanMX* | | This Study |
| AKY9069 | *MATα HMR****a****e** ade2-1 his3-11,15 leu2-3,112 trp1-1 ura3-1 can1-100 eco1-1* | | This Study |
| AKY9070 | *MATα HMR****a****e** ade2-1 his3-11,15 leu2-3,112 trp1-1 ura3-1 can1-100 eco1-1 sas2*Δ*::KanMX* | | This Study |
| AKY1744 | *MATα HMR****a****e** ade2-1 his3-11,15 leu2-3, 112 ura3-1 trp1-1 hht1-hhf1*Δ*::LEU2 hht2-hhf2*Δ*::HIS3* pPK189 | | [4]^1^ |
| AKY1692 | *MAT****a*** *HMR*Δ*::ADE2 ade2-1 his3-11,15 leu2-3, 112 ura3-1 trp1-1 can1-100 hht1-hhf1*Δ*::LEU2 hht2-hhf2*Δ*::HIS3* pPK189 | | [4]^1^ |
| AKY2809 | *MAT****a*** *HMR*Δ*::ADE2 ade2-1 his3-11,15 leu2-3, 112 ura3-1 trp1-1 can1-100 hht1-hhf1*Δ*::LEU2 hht2-hhf2*Δ*::HIS3 cac1*Δ*::KanMX* pPK189 | | [4]^1^ |
| AKY2631 | *MAT****a*** *HMR*Δ*::ADE2 ade2-1 his3-11,15 leu2-3, 112 ura3-1 trp1-1 can1-100 hht1-hhf1*Δ*::LEU2 hht2-hhf2*Δ*::HIS3 rtt106*Δ*::KanMX* pPK189 | | This Study^1^ |
| AKY2581 | *MAT****a*** *HMR*Δ*::ADE2 ade2-1 his3-11,15 leu2-3, 112 ura3-1 trp1-1 can1-100 hht1-hhf1*Δ*::LEU2 hht2-hhf2*Δ*::HIS3 hif1*Δ*::NatMX* pPK189 | | This Study^1^ |
| AKY2633 | *MAT****a*** *HMR*Δ*::ADE2 ade2-1 his3-11,15 leu2-3, 112 ura3-1 trp1-1 can1-100 hht1-hhf1*Δ*::LEU2 hht2-hhf2*Δ*::HIS3 hat1*Δ*::NatMX* pPK189 | | This Study^1^ |
| AKY3706 | *MAT****a*** *HMR*Δ*::ADE2 ade2-1 his3-11,15 leu2-3, 112 ura3-1 trp1-1 can1-100 hht1-hhf1*Δ*::LEU2 hht2-hhf2*Δ*::HIS3 rtt109*Δ*::KanMX* pPK189 | | [4]^1^ |
| AKY3406 | *MAT****a*** *HMR*Δ*::ADE2 ade2-1 his3-11,15 leu2-3, 112 ura3-1 trp1-1 can1-100 hht1-hhf1*Δ*::LEU2 hht2-hhf2*Δ*::HIS3 sas2*Δ*::NatMX* pPK189 | | This Study^1^ |
| AKY2811 | *MAT****a*** *HMR*Δ*::ADE2 ade2-1 his3-11,15 leu2-3, 112 ura3-1 trp1-1 can1-100 hht1-hhf1*Δ*::LEU2 hht2-hhf2*Δ*::HIS3 asf1*Δ*::KanMX* pPK189 | | [4]^1^ |
| AKY3407 | *MAT****a*** *HMR*Δ*::ADE2 ade2-1 his3-11,15 leu2-3, 112 ura3-1 trp1-1 can1-100 hht1-hhf1*Δ*::LEU2 hht2-hhf2*Δ*::HIS3 dot1*Δ*::NatMX* pPK189 | | [5] |
| AKY5158 | *MAT****a*** *his3*Δ*1 leu2*Δ*200 met15*Δ*0 ura3*Δ*0 ASF1-GFP(S65T)-HIS3MX* | | [6] |
| AKY6305 | *MAT****a*** *his3*Δ*1 leu2*Δ*200 met15*Δ*0 ura3*Δ*0 ASF1-GFP(S65T)-HIS3MX* pAK1185 | | This study |
| AKY6307 | *MAT****a*** *his3*Δ*1 leu2*Δ*200 met15*Δ*0 ura3*Δ*0 ASF1-GFP(S65T)-HIS3MX* pAK1166 | | This study |
| AKY6517 | *MAT****a*** *his3*Δ*1 leu2*Δ*200 met15*Δ*0 ura3*Δ*0 ASF1-GFP(S65T)-HIS3MX rtt109*Δ*::KanMX* pAK1185 | | This study |
| AKY6106 | *MAT****a*** *his3*Δ*1 leu2*Δ*200 met15*Δ*0 ura3*Δ*0 ASF1-GFP(S65T)-HIS3MX pol30*Δ*::hisG* pAK1168 | | This study |
| AKY5164 | *MAT****a*** *his3*Δ*1 leu2*Δ*200 met15*Δ*0 ura3*Δ*0 RTT106-GFP(S65T)-HIS3MX* | | [6] |
| AKY5448 | *MATα met15*Δ*0 can1*Δ*::STE2pr-LEU2 ASF1-mCherry-URA3 RTT106-GFP(S65T)-HIS3MX* | | This study |
| AKY6212 | *MAT****a*** *his3*Δ*1 leu2*Δ*200 met15*Δ*0 ura3*Δ*0 RTT106-GFP(S65T)-HIS3MX* pAK1166 | | This study |
| AKY6244 | *MATα met15*Δ*0 can1*Δ*::STE2pr-LEU2 ASF1-mCherry-URA3 RTT106-GFP(S65T)-HIS3MX rtt109*Δ*::KanMX* | | This study |
| AKY8036 | *MAT****a*** *his3*Δ*1 leu2*Δ*200 met15*Δ*0 ura3*Δ*0 ASF1-GFP(S65T)-HIS3MX rtt101*Δ*::KanMX* pAK1185 | | This study |
| AKY6813 | *MAT****a*** *his3*Δ*1 leu2*Δ*200 lys2*Δ*0 ura3*Δ*0 SAS5-yEmCitrine-spHIS5* | | This Study |
| AKY6815 | *MAT****a*** *his3*Δ*1 leu2*Δ*200 lys2*Δ*0 ura3*Δ*0 SAS5-yEmCitrine-spHIS5 cac1*Δ*::KanMX* | | This Study |
| AKY6817 | *MAT****a*** *his3*Δ*1 lys2Δ0 ura3*Δ*0 SAS5-yEmCitrine-spHIS5 asf1*Δ*::KanMX* | | This Study |
| AKY6819 | *MAT****a*** *his3*Δ*1 leu2*Δ*200 lys2*Δ*0 ura3*Δ*0 SAS5-yEmCitrine-spHIS5 rtt106*Δ*::KanMX* | | This Study |
| AKY9277 | *MAT****a*** *his3*Δ*1 leu2*Δ*200 lys2*Δ*0 ura3*Δ*0 SAS5-yEmCitrine-spHIS5 rtt109*Δ*::KanMX* | | This Study |
| AKY1860 | *MATα HMR****a****e** sir2*Δ*::HIS3 his3-11,15 leu2-3,112 trp1-1 ura3-1 lys2*Δ *can1-100* | | This Study |
| AKY8786 | *MATα HMR****a****e** sir2*Δ*::TRP1 cac1*Δ*::KanMX ade2-1 his3-11,15 leu2-3,112 trp1-1 ura3-1 can1-100* | | This Study |
| ^1^Parental Strains used for this study. See S2 Table for description of plasmids that were introduced into the above strains during strain construction or for experiments described in text. | | | |
|  |  |  |  |

**References**

1. Brachmann CB, Davies A, Cost GJ, Caputo E, Li J, Hieter P, et al. Designer deletion strains derived from Saccharomyces cerevisiae S288C: a useful set of strains and plasmids for PCR-mediated gene disruption and other applications. Yeast. 1998;14(2):115-32.

2. Miller A, Chen J, Takasuka TE, Jacobi JL, Kaufman PD, Irudayaraj JM, et al. Proliferating cell nuclear antigen (PCNA) is required for cell cycle-regulated silent chromatin on replicated and nonreplicated genes. J Biol Chem. 2010;285(45):35142-54.

3. Meijsing SH, Ehrenhofer-Murray AE. The silencing complex SAS-I links histone acetylation to the assembly of repressed chromatin by CAF-I and Asf1 in Saccharomyces cerevisiae. Genes Dev. 2001;15(23):3169-82.

4. Miller A, Yang B, Foster T, Kirchmaier AL. Proliferating cell nuclear antigen and ASF1 modulate silent chromatin in Saccharomyces cerevisiae via lysine 56 on histone H3. Genetics. 2008;179(2):793-809.

5. Yang B, Britton J, Kirchmaier AL. Insights into the impact of histone acetylation and methylation on Sir protein recruitment, spreading, and silencing in Saccharomyces cerevisiae. J Mol Biol. 2008;381(4):826-44.

6. Huh WK, Falvo JV, Gerke LC, Carroll AS, Howson RW, Weissman JS, et al. Global analysis of protein localization in budding yeast. Nature. 2003;425(6959):686-91.
