# Supplementary material for: CAF-1 and Rtt101p function within the replication-coupled chromatin assembly network to promote H4 K16ac, preventing ectopic silencing
Source: PLoS Genet. 2020 Dec 7;16(12):e1009226. doi: 10.1371/journal.pgen.1009226 (PMC7746308; doi:10.1371/journal.pgen.1009226)
Supplement: S2 Table — (DOCX) [file pgen.1009226.s002.docx]

**S2 TABLE. Plasmids used in this study.**

| **Plasmid** | **Description** | **Source** |
| --- | --- | --- |
| pRS415 | *ARS/CEN/LEU2* | [1] |
| pPK189 | *HHT2-HHF2 ARS/CEN/URA3* | P. Kaufman |
| pMP3 | *HHT2-HHF2 ARS/CEN/TRP1* | [2] |
| pAK972 | *H3/H4 K16R ARS/CEN/TRP1* | [3] |
| pAK965 | *H3 K56R ARS/CEN/TRP1* | [3] |
| pAK1011 | *H3 K122A/H4 ARS/CEN/TRP1* | This Study |
| pAK1326 | *H3 K122R/H4 ARS/CEN/TRP1* | This Study |
| pAK1327 | *H3 K122Q/H4 ARS/CEN/TRP1* | This Study |
| pAK1314 | *H3 K122,125R/H4 ARS/CEN/TRP1* | This Study |
| pAK1323 | *H3 K121,122,125R/H4 ARS/CEN/TRP1* | This Study |
| pRS416 | *ARS4/CEN6/URA3* | [1] |
| pAK1223 | *CAC1 ARS/CEN/URA3* | [4] |
| pAK1308 | *cac1 S238, 503D ARS/CEN/URA3* | [4] |
| pAK1309 | *cac1 S238, 503A ARS/CEN/URA3* | [4] |
| pAK1328 | *cac1 S501, 503D ARS/CEN/URA3* | [4] |
| pAK1329 | *cac1 S501, S503A ARS/CEN/URA3* | [4] |
| AE778 | *6myc-SAS2 ARS/CEN/LEU2* | [5] |
| pAK1347 | *6myc-SAS2-M1 ARS/CEN/LEU2* | This Study |
| pAK1185 | *CAC1-mCherry ARS/CEN/URA3* | [4] |
| pAK1128 | *mCherry-KanMX* cassette | [4] |
| pAK1166 | *SPC29-mCherry ARS/CEN/LEU2* | [4] |
| pAK1168 | *POL30-mCherry ARS/CEN/LEU2* | This Study |
| pFA6::KanMX4 | *KanMX cassette* | [6] |
| pFA6::NatMX4 | *NatMX cassette* | [7] |
| pBL243 | *POL30*Δ*::hisGURA3hisG* | [8] |

**References**

1. Christianson TW, Sikorski RS, Dante M, Shero JH, Hieter P. Multifunctional yeast high-copy-number shuttle vectors. Gene. 1992;110(1):119-22.

2. Kelly TJ, Qin S, Gottschling DE, Parthun MR. Type B histone acetyltransferase Hat1p participates in telomeric silencing. Mol Cell Biol. 2000;20(19):7051-8.

3. Miller A, Yang B, Foster T, Kirchmaier AL. Proliferating cell nuclear antigen and ASF1 modulate silent chromatin in Saccharomyces cerevisiae via lysine 56 on histone H3. Genetics. 2008;179(2):793-809.

4. Young TJ, Cui Y, Irudayaraj J, Kirchmaier AL. Modulation of Gene Silencing by Cdc7p via H4 K16 Acetylation and Phosphorylation of Chromatin Assembly Factor CAF-1 in Saccharomyces cerevisiae. Genetics. 2019;211(4):1219-37.

5. Meijsing SH, Ehrenhofer-Murray AE. The silencing complex SAS-I links histone acetylation to the assembly of repressed chromatin by CAF-I and Asf1 in Saccharomyces cerevisiae. Genes Dev. 2001;15(23):3169-82.

6. Wach A, Brachat A, Pohlmann R, Philippsen P. New heterologous modules for classical or PCR-based gene disruptions in Saccharomyces cerevisiae. Yeast. 1994;10(13):1793-808.

7. Goldstein AL, McCusker JH. Three new dominant drug resistance cassettes for gene disruption in Saccharomyces cerevisiae. Yeast. 1999;15(14):1541-53.

8. Ayyagari R, Impellizzeri KJ, Yoder BL, Gary SL, Burgers PM. A mutational analysis of the yeast proliferating cell nuclear antigen indicates distinct roles in DNA replication and DNA repair. Mol Cell Biol. 1995;15(8):4420-9.
