# Supplementary material for: CAF-1 and Rtt101p function within the replication-coupled chromatin assembly network to promote H4 K16ac, preventing ectopic silencing
Source: PLoS Genet. 2020 Dec 7;16(12):e1009226. doi: 10.1371/journal.pgen.1009226 (PMC7746308; doi:10.1371/journal.pgen.1009226)
Supplement: S3 Table — (DOCX) [file pgen.1009226.s003.docx]

**S3 TABLE. Oligos used in this study.**

| **Region** | **Oligos** | **Application** |
| --- | --- | --- |
| *cac1*Δ*::KanMX* | oALK507 5' TAGTGAACCTCAAGACAGAAGAGAATCGAAAGGAAAAGGGAAACGT  ACGCTGCAGGTCGAC  oALK508 5' CAGTTTATCTGTATGTTTCTATATACTAAAGATCCGTTCAAGATCGAT  GAATTCGAGCTCG | Gene Disruption |
| *cac1Δ::KanMX* | oALK630 5’ GAATTTCTCTAGATACACCAAC | Screen for Knockout of *CAC1* |
| *rtt101*Δ*::KanMX* | oALK1493 5' CAGATTTACCTTCATCCTGCA oALK1494 5' TGTTCAGATAGGAGCGAAAGA | Gene Disruption |
| *rtt101*Δ*::KanMX* | oALK1518 5' CCTTCGTATCATAATC | Screen for Knockout of *RTT101* |
| *rtt106*Δ*::KanMX* | oALK587 5' GGCAGTTGCTTTCAGATGCAT oALK588 5' CCTGGAAACTGCCGTTGAAAG | Gene Disruption |
| *rtt106*Δ*::KanMX* | oALK589 5' GACCCCATAGGAACCATTTCT | Screen for Knockout of *RTT106* |
| *hif1*Δ*::KanMX* | oALK601 5' TAACATCGGCGATGAGGTCTCCTTCTAGAAGTAACAGAATCGTACGC  TGCAGGTCGAC  oALK602 5' TATACATTACGTATGTAGGTTGCTACATTTTACACAAAACTAATCGAT  GAATTCGAGCTCG | Gene Disruption |
| *hif1*Δ*::KanMX* | oALK586 5' TCACCCGGCGCAGTATTTCTT | Screen for Knockout of *HIF1* |
| *hat1*Δ*::KanMX* | oALK599 5' TCCCTTAGTTCGACAGATTGC oALK600 5' TTCGCTTTAGAACCGCTACCATGG | Gene Disruption |
| *hat1*Δ*::KanMX* | oALK611 5' GGATCAATCCCAATAGATGAGCA | Screen for Knockout of *HAT1* |
| *mms1*Δ*::KanMX* | oALK1577 5' GGCTAAGTAGCGCCAGGATAT oALK1578 5' ACCATCACCAACGATTACCA | Gene Disruption |
| *mms1*Δ*::KanMX* | oALK1579 5' ACGGTTTTCTTTGGTGGAGCCA | Screen for Knockout of *MMS1* |
| *mms22*Δ*::KanMX* | oALK1580 5' CGCATGCATGAAGAATTTCG oALK1581 5' TGCCGCATTTGGGTACTTAT | Gene Disruption |
| *mms22*Δ*::KanMX* | oALK1582 5' CGGGTCCCTTCACTACCCAATG | Screen for Knockout of *MMS22* |
| *ctf1*Δ*::KanMX* | oALK1722 5’ TTTGAACCGTCGCTGTACCAC | Screen for Knockout of *CTF1* |
| *HHT2* | oALK706 5' GTTTTGTGACTTCCACTTTGGCCCT | Sequencing |
| H3 K122R | oALK1620 5' AAGCGTGTTACTATCCAAAAGAGGGATATCAAATTGGCCAGAAGA oALK1621 5' TCTTCTGGCCAATTTGATATCCCTCTTTTGGATAGTAACACGCTT | Site-directed mutagenesis |
| H3 K122Q | oALK1622 5' AAGCGTGTTACTATCCAAAAGCAGGATATCAAATTGGCCAGAAGA oALK1623 5' TCTTCTGGCCAATTTGATATCCTGCTTTTGGATAGTAACACGCTT | Site-directed mutagenesis |
| H3 K122A | oALK832 5' CTATCCAAAAGGCGGATATCAAATTGG oALK833 5' CCAATTTGATATCCGCCTTTTGGATAG | Site-directed mutagenesis |
| *H3 K122, 125R* | oALK1569 5' GTGTTACTATCCAAAAGAGGGATATCAG  ATTGGCCAGAAGACTAA  oALK1570 5' TTAGTCTTCTGGCCAATCTGATATCCCTC  TTTTGGATAGTAACAC | Site-directed mutagenesis |
| *H3 K121, 122, 125R* | oALK1567 5' GCGTGTTACTATCCAAAGGAGGGATATC  AGATTGGCCAGAAGACTA  oALK1568 5' TAGTCTTCTGGCCAATCTGATATCCCTCC  TTTGGATAGTAACACGC | Site-directed mutagenesis |
| *NatMX* | oALK217 5' TTCGTCGTCGGGGAACACCTT | Reverse Screening Primer |
| *KanMX* | oALK72 5' CTGCAGCGAGGAGCCGTAAT | Reverse Screening Primer |
| *HMR****a****e*** | oALK1299 5’ ATTATATTGCACAAACA oALK1300 5’ TCGCCTACCTTCTTGAA | Screen for *HMR****a****e*** |
| *MAT****a***  *MATα* | oALK303 5’ CTCGCTGAAGAATGGCACG  oALK304 5’ GTTCTTAGCTTGTACCAGAGGAAGC  oALK171 5’ GGATGATATTTGTAGTATGGCGG | Screen for Mating Type |
| *e*** | oALK261 5’ CCCGTCCAAGTTATGAGCTTAATCT  oALK262 5’ GGAGTCTTAATTTCCCTGATTTTAGTTTAG | ChIP |
| ***a1*** | oALK270 5’ TTTAGAAGAAAGCAAAGCCTTAATTCC  oALK271 5’ CTTGAAGTGGAGTAATGCCACATT | ChIP |
