# Supplementary figures and images for: CAF-1 and Rtt101p function within the replication-coupled chromatin assembly network to promote H4 K16ac, preventing ectopic silencing
Source: PLoS Genet. 2020 Dec 7;16(12):e1009226. doi: 10.1371/journal.pgen.1009226 (PMC7746308; doi:10.1371/journal.pgen.1009226)

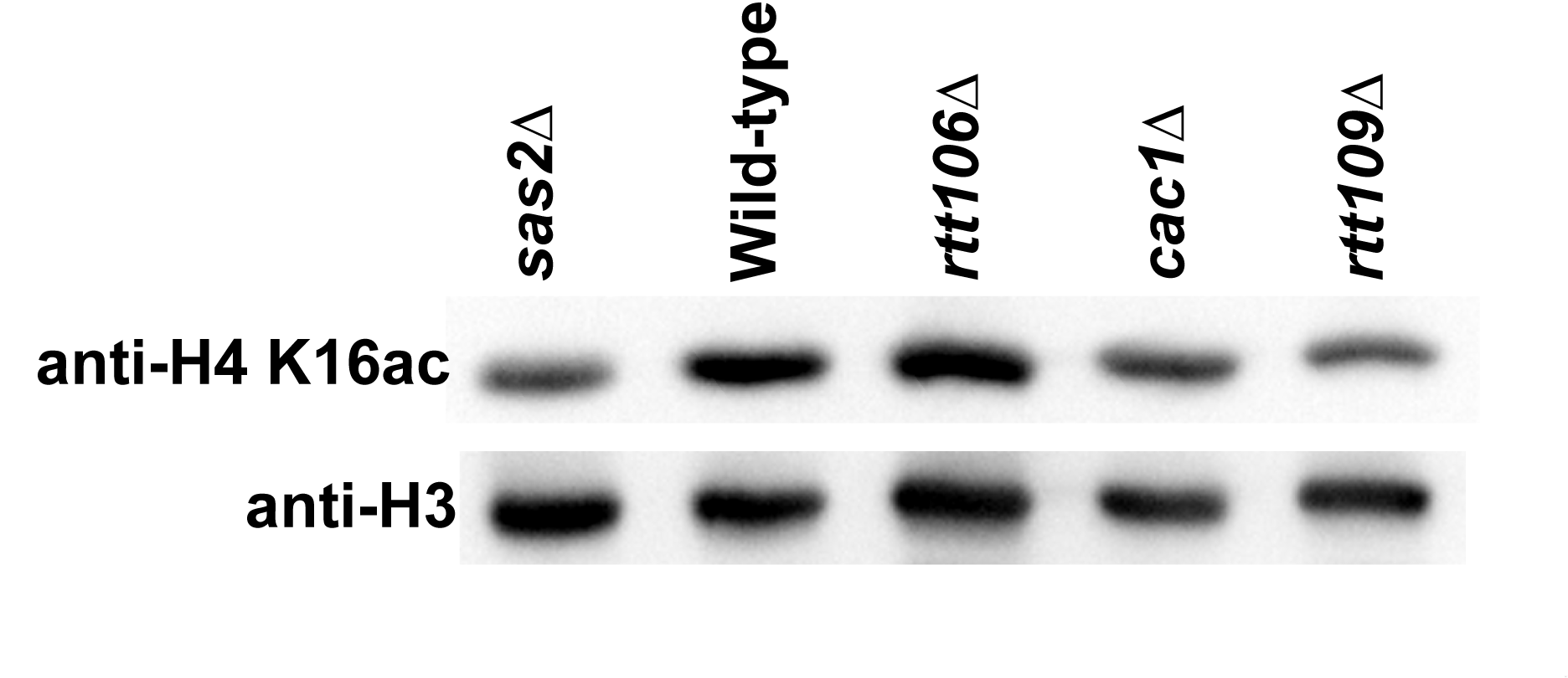

Supplement: S1 Fig — Immunoblot analysis of H4 K16ac and H3 levels in chromatin fractions isolated from indicated genotypes. Immunoblot shown is representative of three biological replicates used to generate quantification data in Table 1. (TIF) [file pgen.1009226.s005.tif]

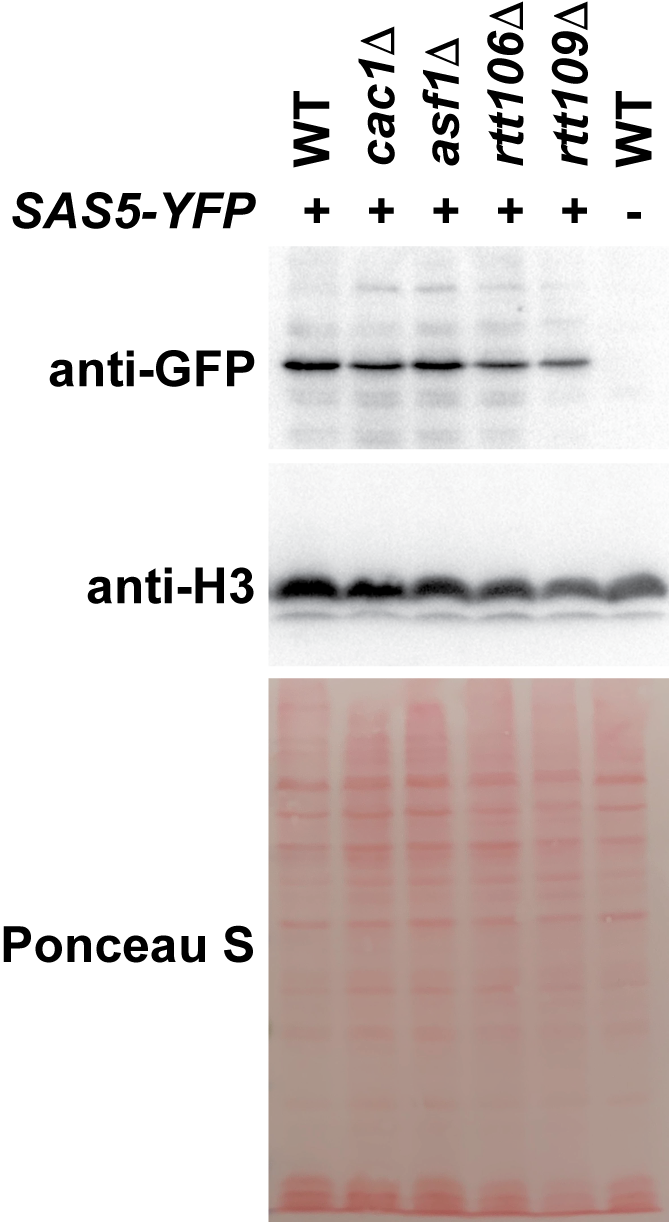

Supplement: S2 Fig — Immunoblot analysis of Sas5-YFP and H3 levels (loading control) in chromatin fractions isolated from indicated genotypes with Ponceau S staining of total protein levels. Immunoblot shown is representative of four biological replicates used to generate quantification data in Table 2. (TIF) [file pgen.1009226.s006.tif]

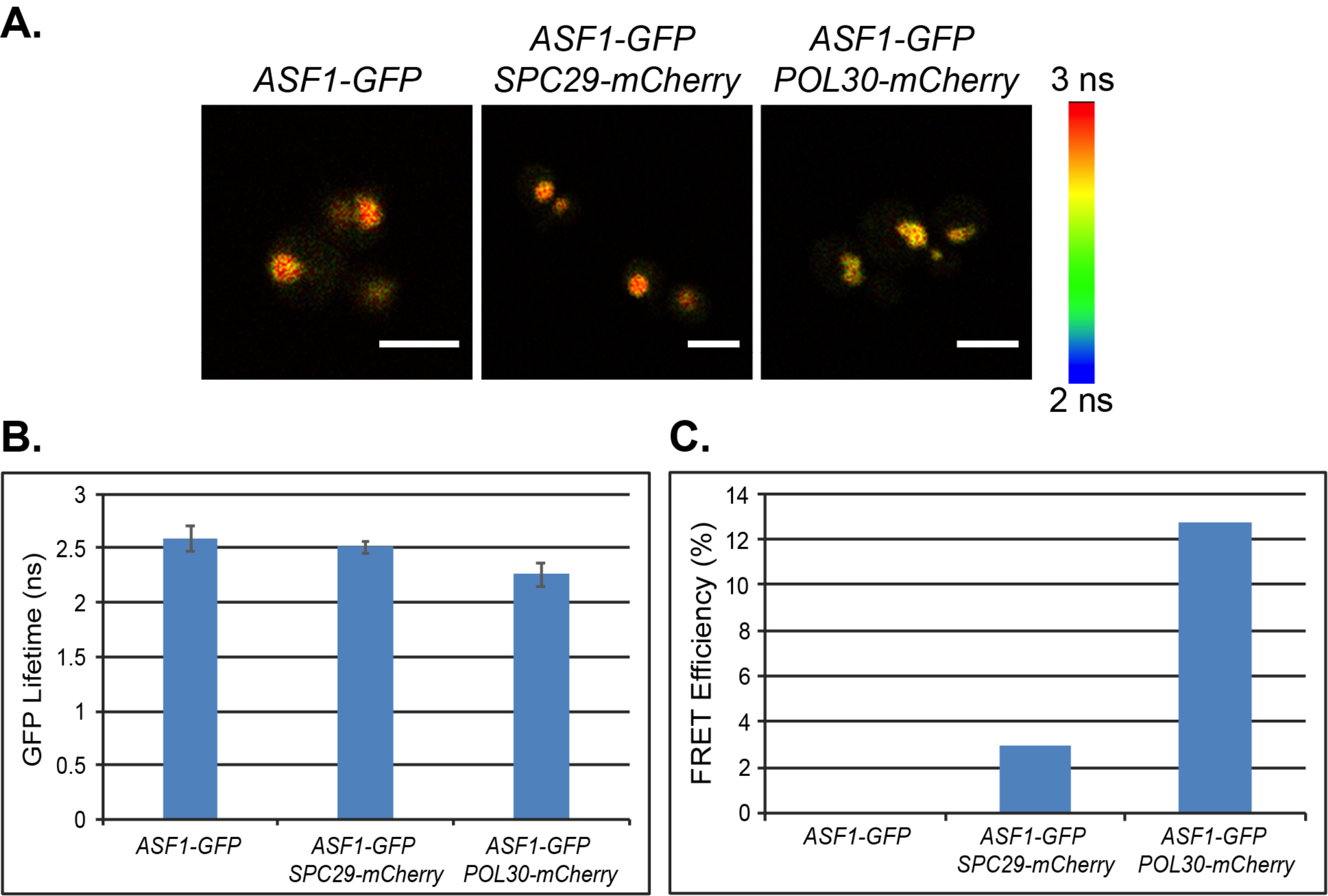

Supplement: S3 Fig — A) Confocal fluorescence lifetime images of GFP in small-budded live cells expressing the fluorescently-tagged proteins as indicated. White scale bars are equivalent to 5μm. FLIM scale bar: 1 nanosecond, blue; 3 nanoseconds, red. B) The average lifetime of GFP in indicated strains. Error bars represent the standard deviation of ten FLIM measurements taken for each genotype. C) FRET efficiency of indicated strains. (TIF) [file pgen.1009226.s007.tif]

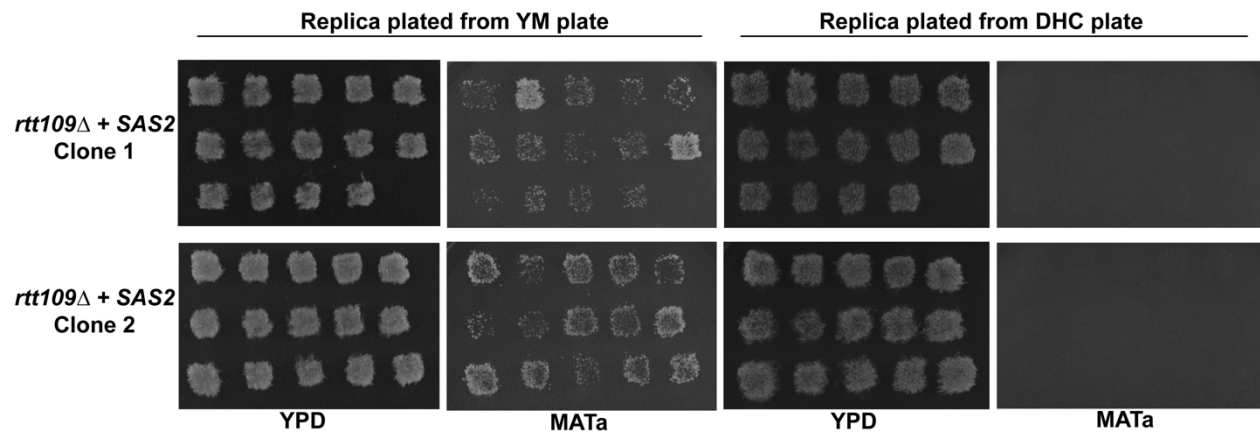

Supplement: S4 Fig — Individual colonies of clones with the indicated genotypes were expanded and grown as individual patches on minimal medium supplemented for auxotrophic markers (YM-LEU) plates at 30°C overnight, then were replica plated onto either a MATa lawn (JRY2726) on minimal medium, or rich medium (YPD), and were grown at 30° for two days. Cells grown on the YM-LEU plate were also replica plated onto a YPD plate containing 300 μM dihydrocoumarin (DHC) to inhibit Sir2p [129–131], grown at 30°C overnight, and then replica plated as above. (PDF) [file pgen.1009226.s008.pdf]

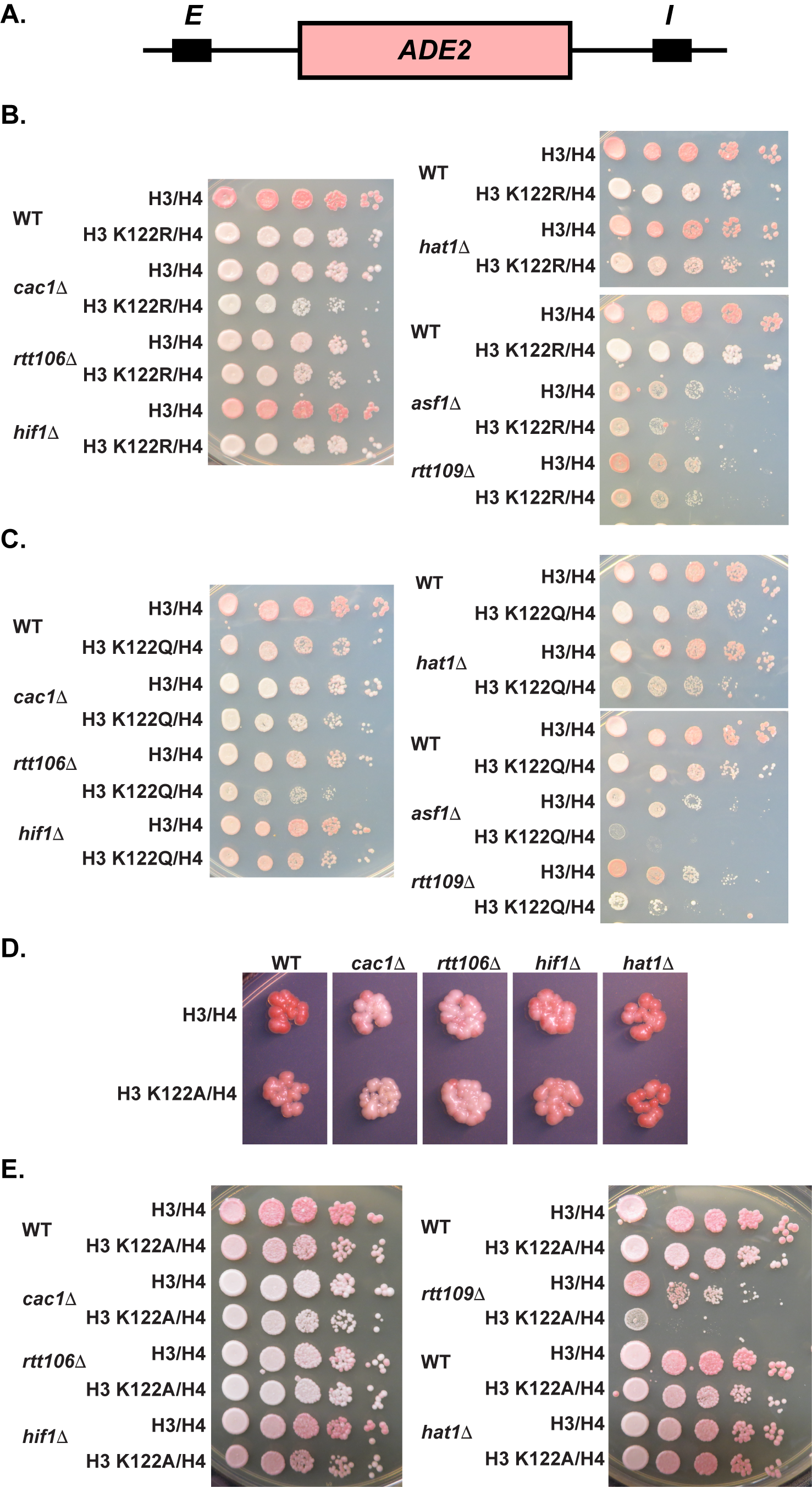

Supplement: S5 Fig — A) Map of HMR::ADE2 Reporter. B-E) Genetic interactions between H3 K122R (B), H3 K122Q (C) or H3 K122A (D and E) and chromatin assembly pathway mutants. Cells with the indicated genotypes were grown in YPD at 30°C overnight, then spotted onto CSM plates in ten-fold serial dilutions, and grown for two days at 30°C. Cells were then incubated at 4°C for four days prior to imaging. (TIF) [file pgen.1009226.s009.tif]

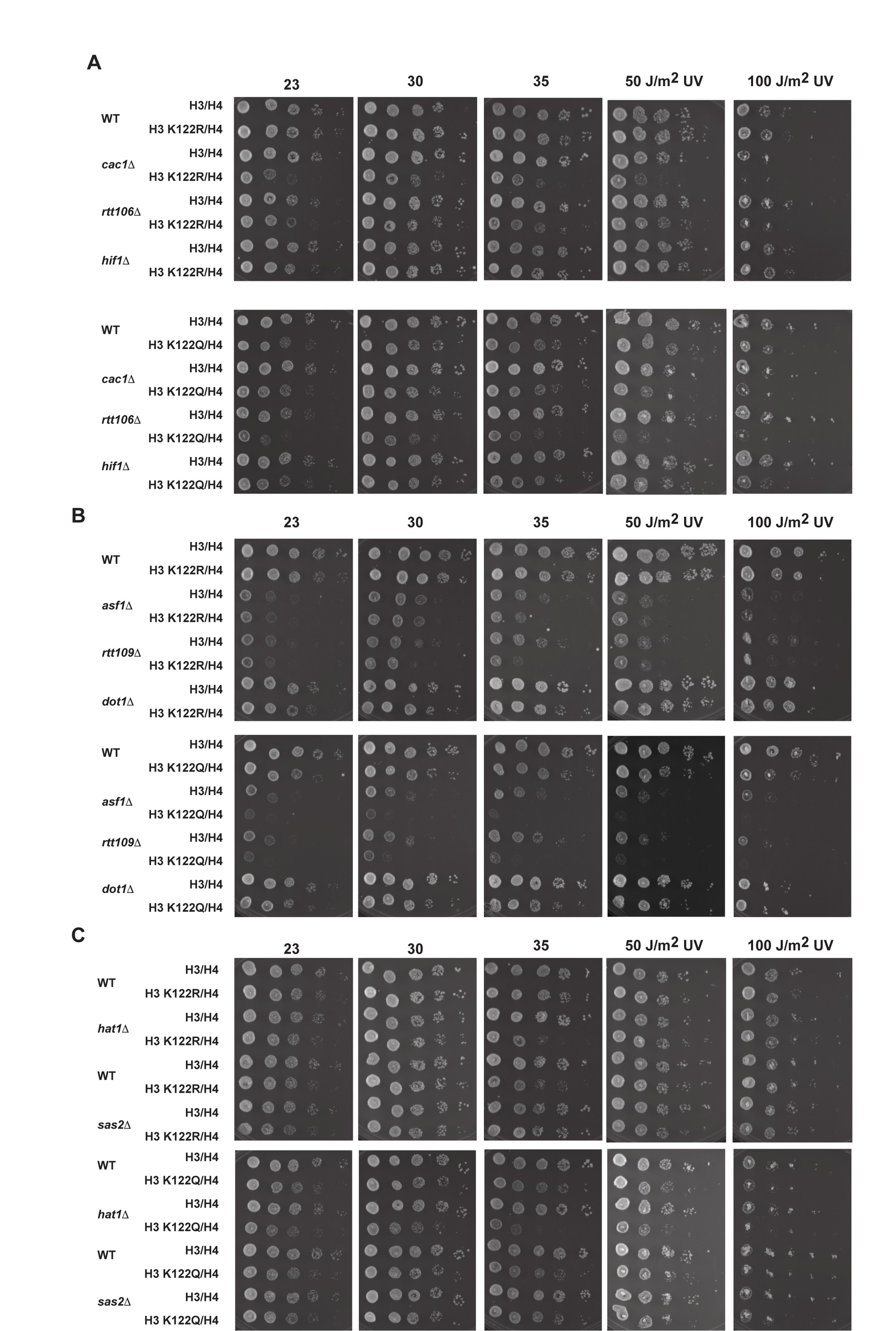

Supplement: S6 Fig — A-C) Genetic interactions between H3 K122R or H3 K122Q and chromatin assembly pathway mutants. Cells with the indicated genotypes were grown in YPD at 30°C overnight, then spotted onto CSM or YPD plates in ten-fold serial dilutions, and grown at the temperature indicated for two days, or were then treated with either 50 or 100 J/m2 of UV light and grown at 30°C for two days. Color images of 30°C plates of some H3 K122Q mutants are shown in S5 Fig. (TIF) [file pgen.1009226.s010.tif]

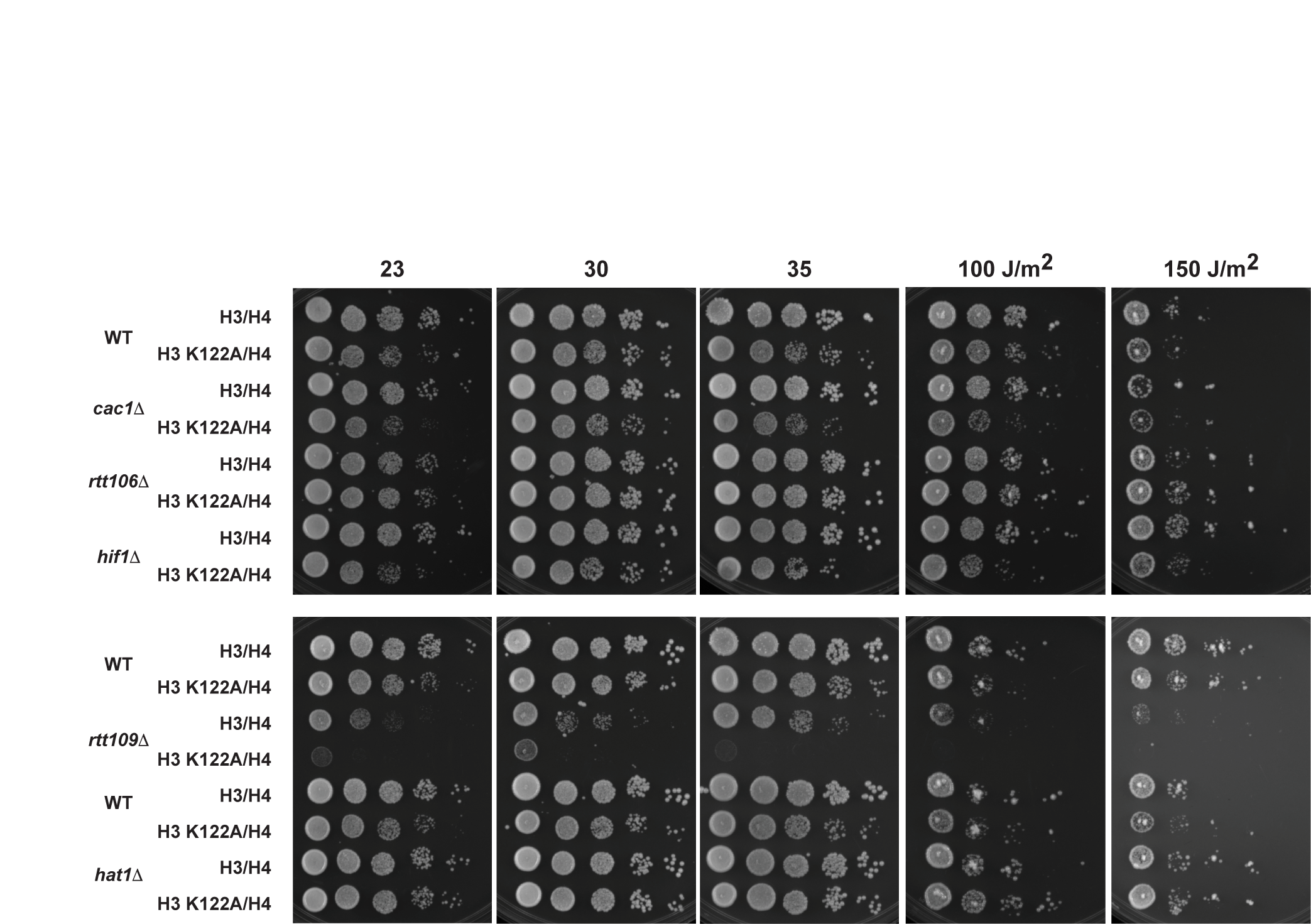

Supplement: S7 Fig — Strains were analyzed as outlined in S6 Fig legend. Color images of 30°C plates are shown in S5 Fig. (TIF) [file pgen.1009226.s011.tif]

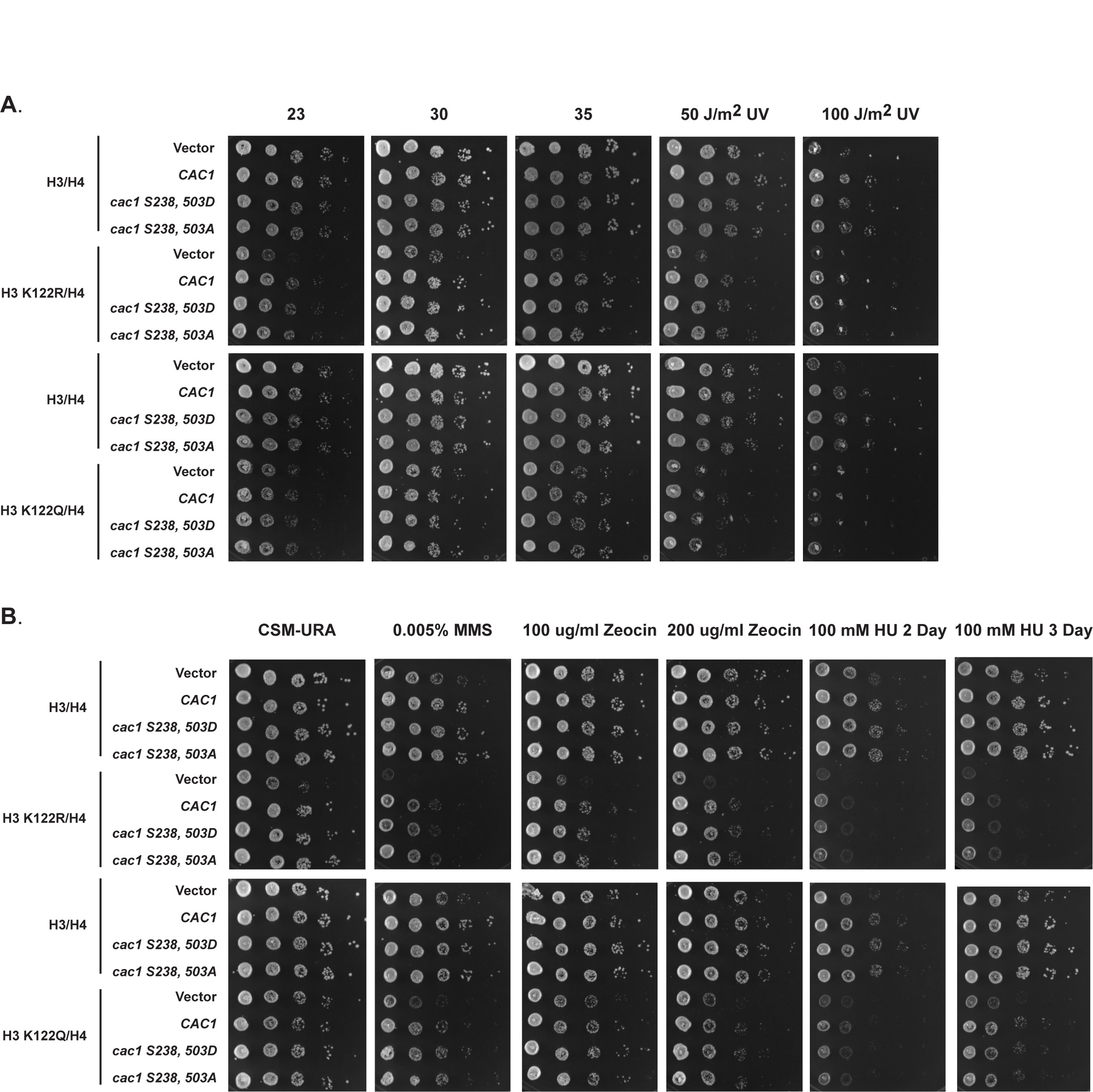

Supplement: S8 Fig — A) Genetic interactions sensitive to temperature or exposure to UV. B) Genetic interactions upon exposure to DNA damaging agents. Yeast with the indicated genotypes were grown in YPD at 30°C overnight, then spotted onto complete supplement medium (CSM) or YPD plates in ten-fold serial dilutions, and grown at the temperature indicated for two days, or were then treated with 50 or 100 J/m2 of UV (A), or the indicated amounts of methyl methanesulfonate (MMS), Zeocin, or hydroxyurea (HU) (B), and grown at 30°C for two days. (TIF) [file pgen.1009226.s012.tif]

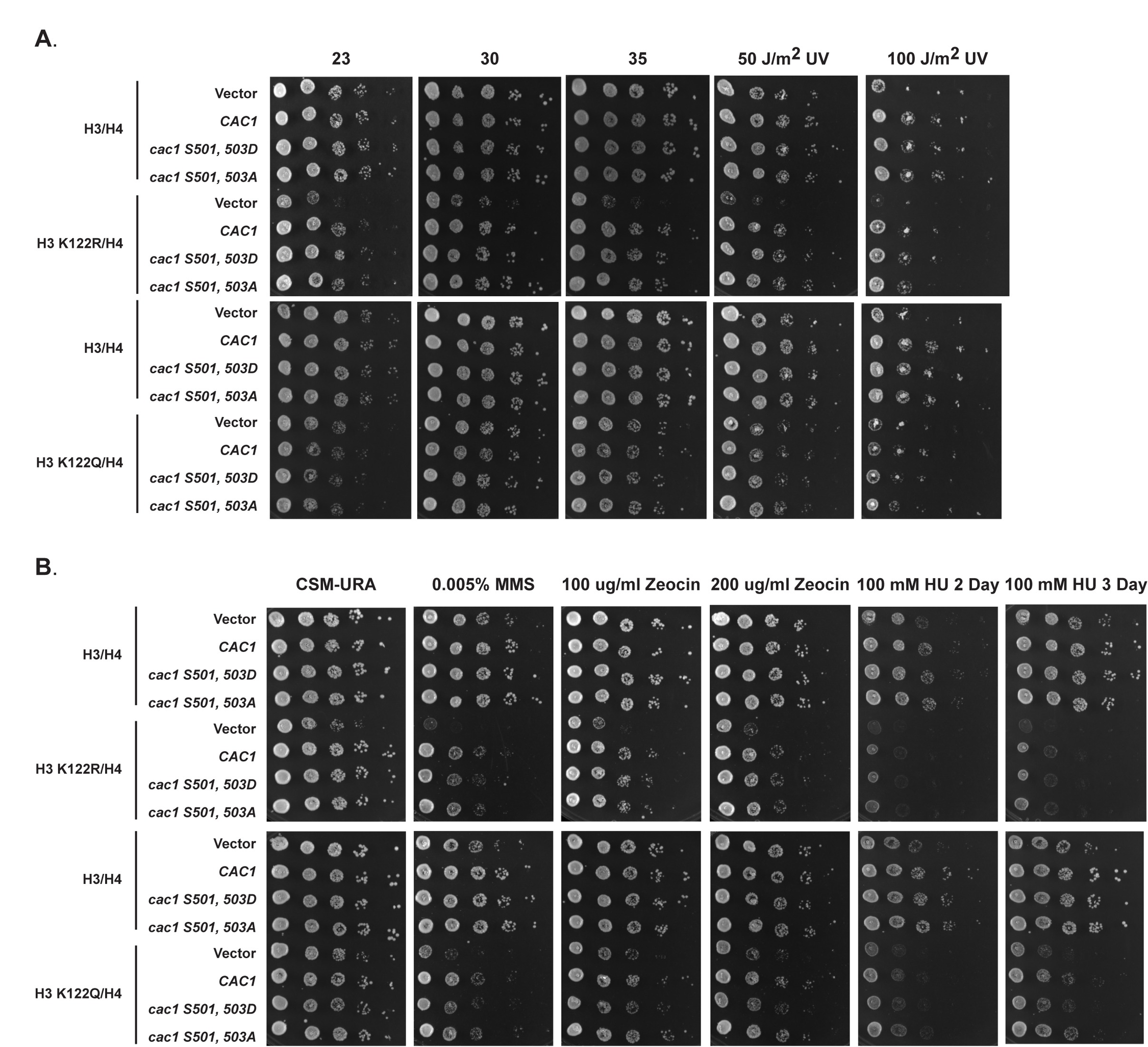

Supplement: S9 Fig — A) Genetic interactions sensitive to temperature or exposure to UV. B) Genetic interactions upon exposure to DNA damaging agents. Yeast were assayed as outlined in S5 Fig. (TIF) [file pgen.1009226.s013.tif]

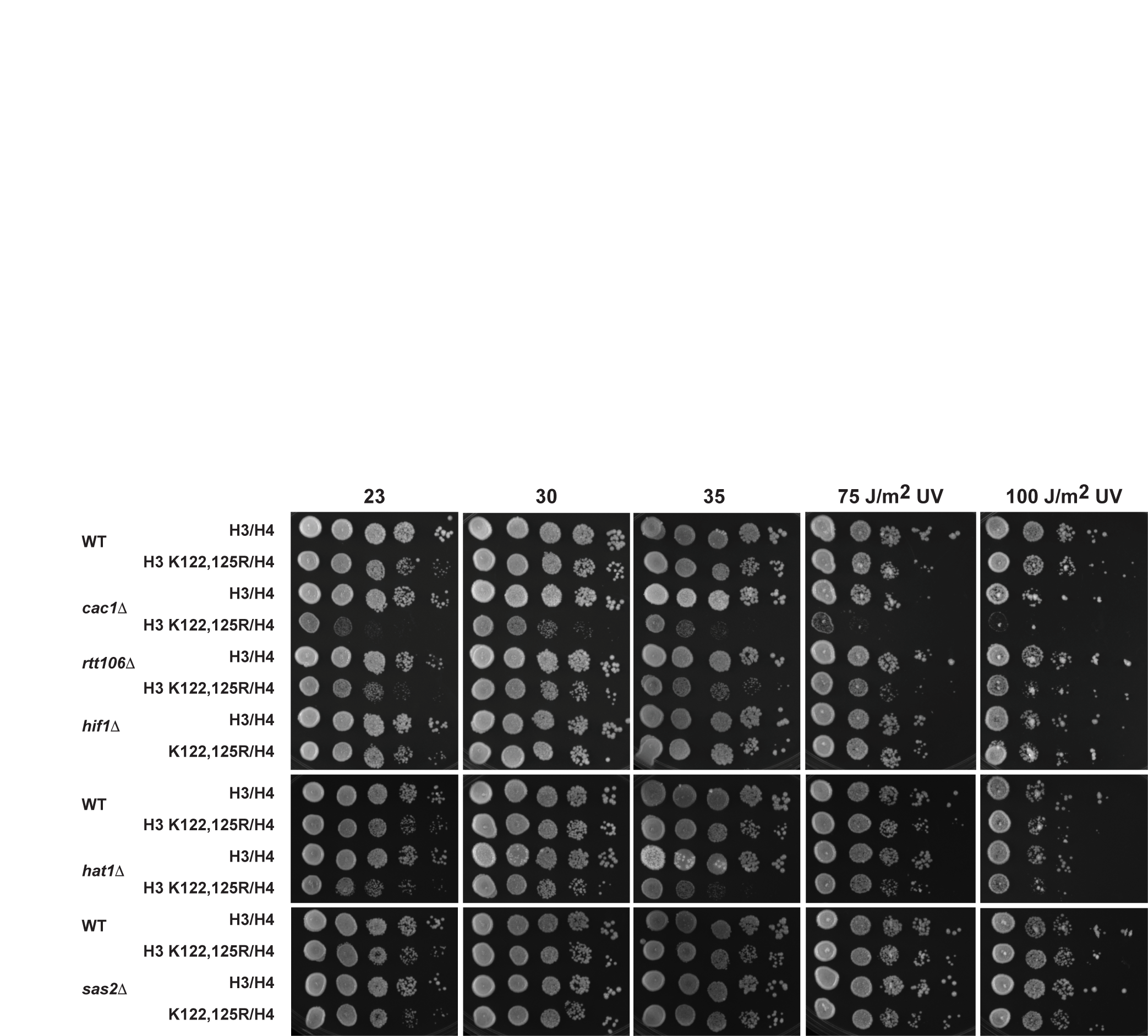

Supplement: S10 Fig — Cells with the indicated genotypes were grown in YPD at 30°C overnight, then spotted onto YPD plates in ten-fold serial dilutions, and grown at the temperature indicated for two days, or were then treated with either 75 or 100 J/m2 of UV light and grown at 30°C for two days. (TIF) [file pgen.1009226.s014.tif]

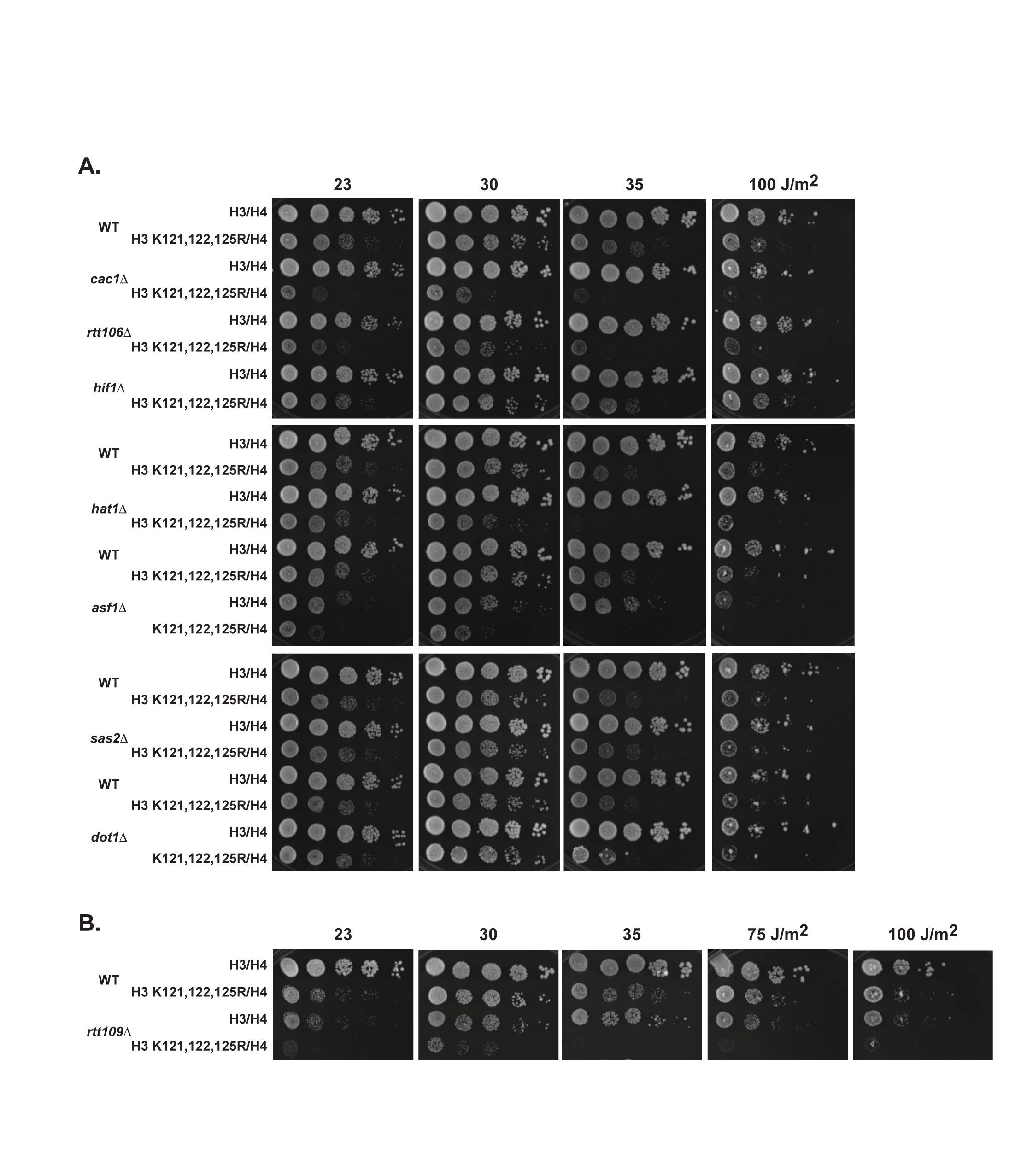

Supplement: S11 Fig — cac1Δ, rtt106Δ, hif1Δ, hat1Δ, asf1Δ, sas1Δ, dot1Δ (A) or rtt109Δ mutants (B) relative to wild-type were assayed as outlined in S7 Fig. (TIF) [file pgen.1009226.s015.tif]

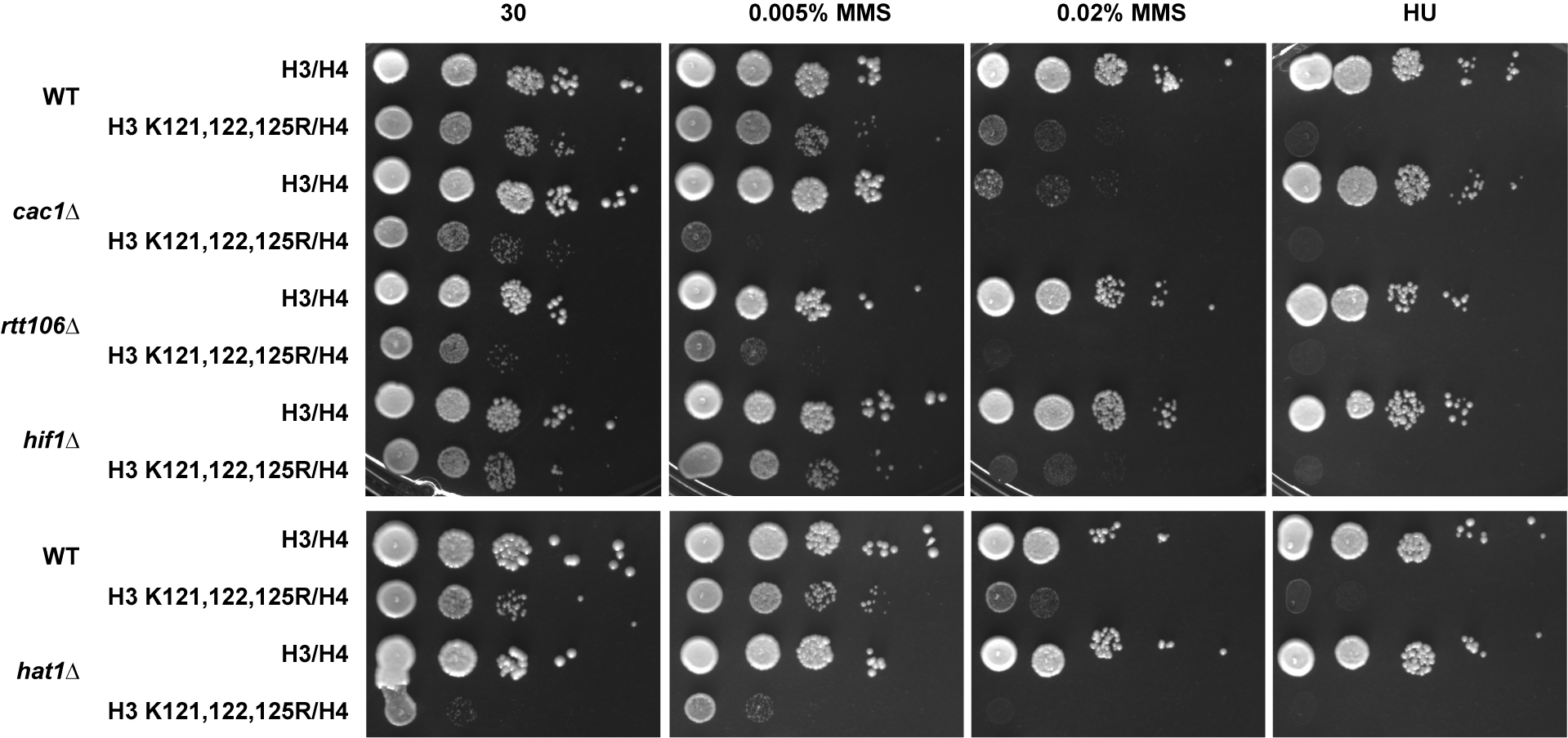

Supplement: S12 Fig — Cells with the indicated genotypes were grown in YPD at 30°C overnight, then spotted onto YPD plates in ten-fold serial dilutions, in the absence or presence of the indicated amounts of MMS or HU at 30°C for two days. (TIF) [file pgen.1009226.s016.tif]

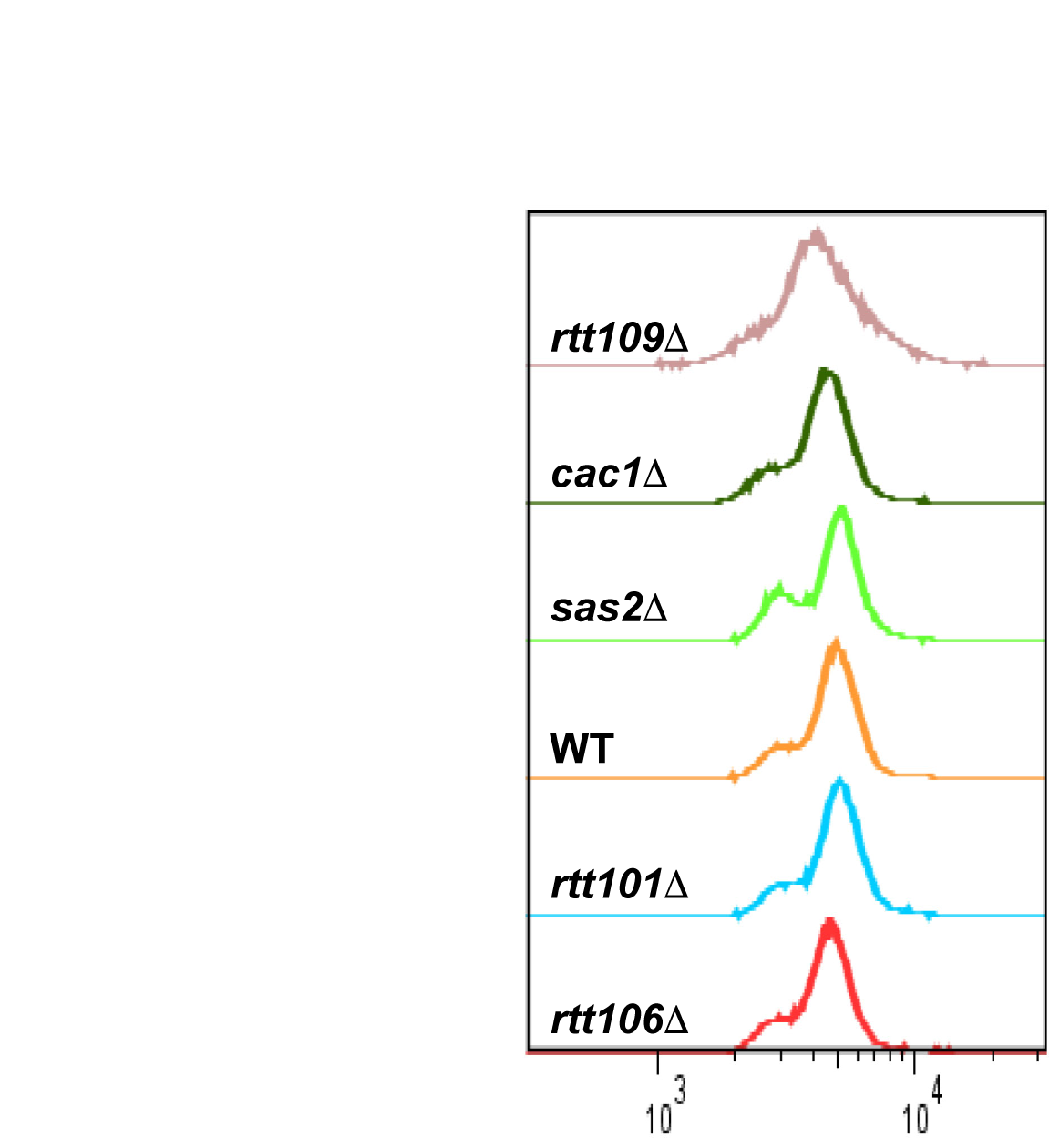

Supplement: S13 Fig — Flow Cytometry. Yeast with the indicated genotypes were grown logarithmically in YPD at 30°C prior to harvesting to assess their cell cycle distribution by Flow Cytometry. (TIF) [file pgen.1009226.s017.tif]
